# Supplementary material for: A seven-transmembrane methyltransferase catalysing N-terminal histidine methylation of lytic polysaccharide monooxygenases
Source: Nat Commun. 2023 Jul 14;14:4202. doi: 10.1038/s41467-023-39875-7 (PMC10349129; doi:10.1038/s41467-023-39875-7)
Supplement: Supplementary file 1 — Supplementary Information [file 41467_2023_39875_MOESM1_ESM.pdf]

Supplementary information for:

A seven-transmembrane methyltransferase catalysing N-terminal histidine methylation of lytic polysaccharide monooxygenases

Tanveer S. Batth[1,*,#], Jonas L. Simonsen[1,2,*], Cristina Hernández-Rollán[3,*], Søren Brander[4], Jens Preben Morth[2], Katja S. Johansen[4], Morten H. H. Nørholm[3, #], Jakob B. Hoof[2, #], and Jesper V. Olsen[1, #].

1) The Novo Nordisk Foundation Center for Protein Research, University of Copenhagen, Denmark

2) Department of Biotechnology and Biomedicine, Technical University of Denmark, Kgs. Lyngby, Denmark.

3) The Novo Nordisk Foundation Center for Biosustainability, Technical University of Denmark, Kongens Lyngby, 2800, Denmark.

4) Department of Geoscience and Natural Resources Management, Copenhagen University, Frederiksberg, DK-1958, Denmark.

*Shared first-authors

#Corresponding authors

Contents

Mass spectrometry data 3
Supplementary Figure 1 – Gene knockout PRM assay 3
***In silico* domain predicition** 4
Supplementary Figure 2 – Secondary structure prediction with PSIPRED and MEMSAT .. 4
Supplementary Figure 3 - Functional validation of NHMT 5
Supplementary Figure 4 – Multiple sequence alignment based prediction 6
Supplementary Figure 5 – *In silico* analysis of the soluble domain 7
Supplementary Figure 6 – Sequence analysis of the 7TM domain shows that it is unique ... 8
Protein expression 9
Supplementary Figure 7 – LsAA9A secretion with the α-MF signal peptide 9
Supplementary Figure 8 – LsAA9A secretion with the Amy signal peptide 10
Supplementary Figure 9 – LsAA9A secretion with its native signal peptide 11
Supplementary Figure 10 – LsAA9A processing with alpha-mating factor signal peptide 12
Supplementary Figure 11 – Tf10A secretion with the Amy signal peptide 13
Supplementary Table 1 – C8V530 pfam predication 14
Supplementary Table 2 - Q5B1W7 pFam predication. 15
Supplementary Table 3 - *Aspergillus nidulans* strains used in this study 16
Supplementary Table 4 - *K. phaffii* strains used in this study. 18
Supplementary Table 5 - Plasmids used in this study 19
Supplementary Table 6 – Oligonucleotides and target sequences used in this study. 22
Supplementary Note 1: Native DNA sequence of LsAA9A used for heterologous expression in *K. phaffii*. 29
Supplementary Note 2: Native DNA sequence of TfAA10A used for heterologous expression in *K. phaffii*. 30
Supplementary Note 3: DNA sequence AN4663 in pBGP1 31
Supplementary References 33

Mass spectrometry data

Supplementary Figure 1 – Gene knockout PRM assay

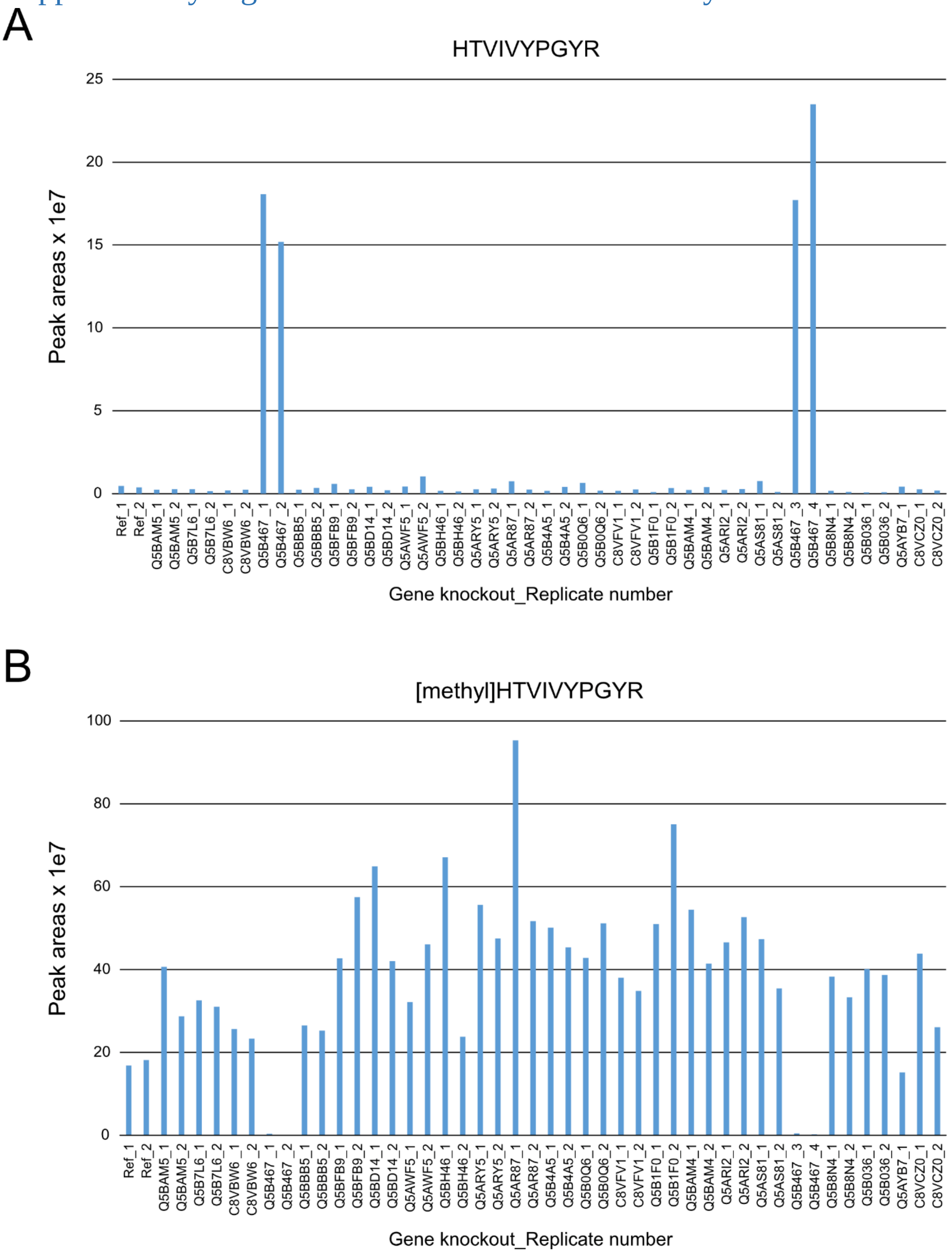

Supplementary Figure 1 – Gene knockout PRM assay. PRM quantification of Q5B428 N-terminal histidine **A)** unmethylated (602.8273 m/z) and **B)** methylated (609.8351 m/z) HTVIVYPGYR peptide for the different gene knockouts shown above with the two biological replicates. Additional biological replicates for AN4663 knockout were performed to confirm the results.

In silico domain predicition

Supplementary Figure 2 – Secondary structure prediction with PSIPRED and MEMSAT

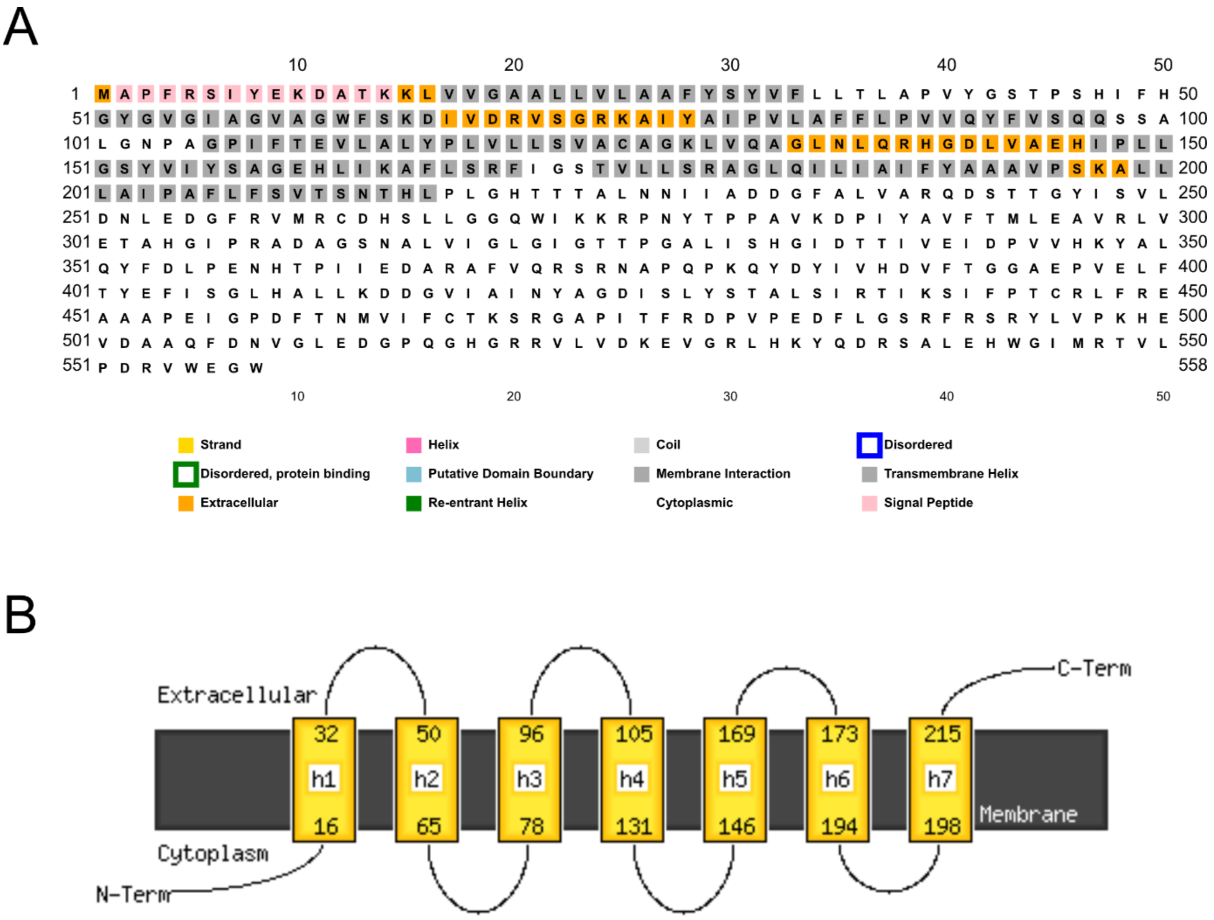

Supplementary Figure 2 – Secondary structure prediction with PSIPRED and MEMSAT. Secondary structure was prediction of the AN4663 protein sequence analyzed using A) PSIPRED shows the predicted secondary structure regions and B) MEMSAT analysis for prediction of transmembrane protein topology of AN4663.

Supplementary Figure 3 - Functional validation of NHMT

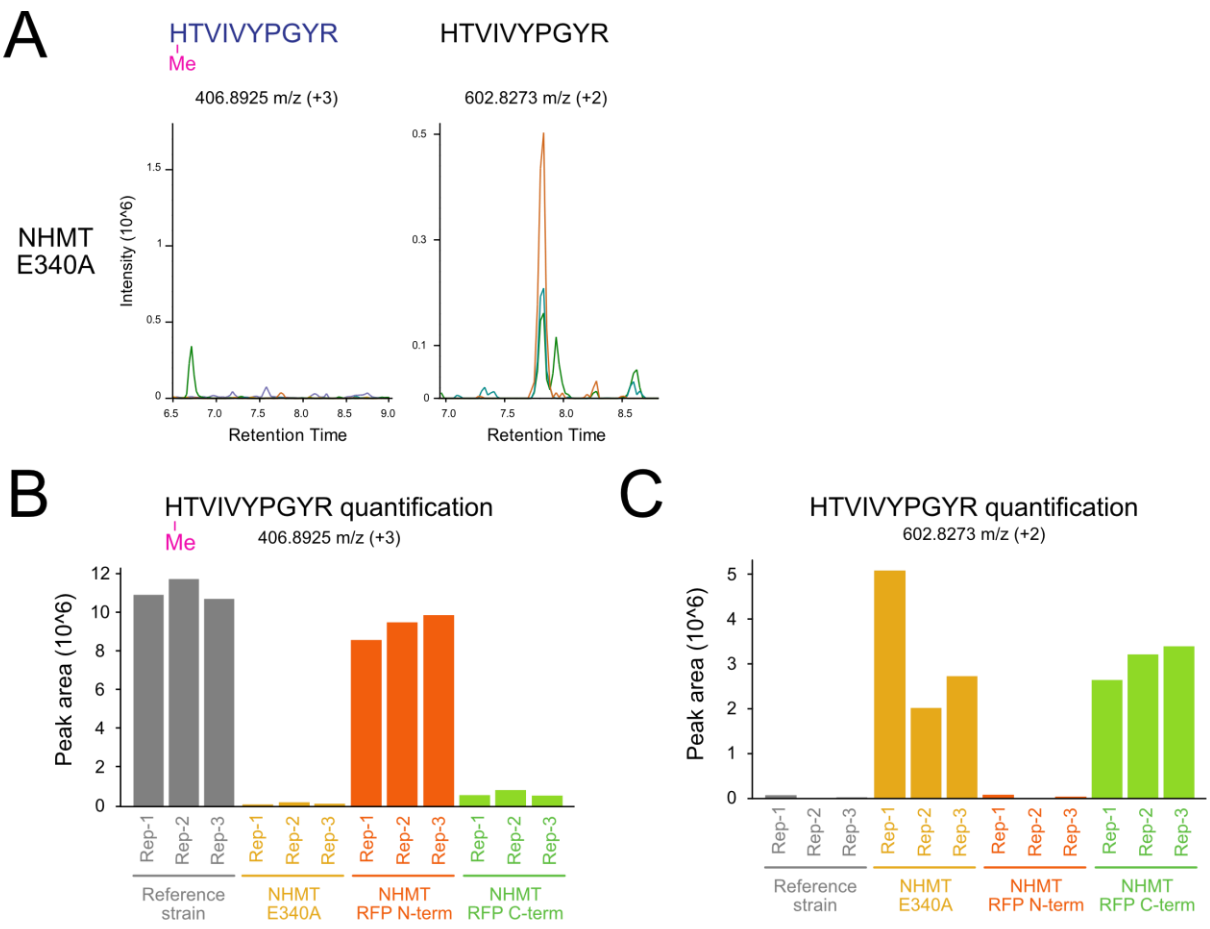

Supplementary Figure 3. Functional validation of NHMT. **A)** PRM chromatogram of the N-terminal histidine methylated and unmethylated HTVIVYPGYR peptide with an NHMT point mutation strain in the SAM catalytic domain at position E340A. **B)** Relative quantification of the N-terminal methylation histidine peptide ([meth]HTVIVPGYR) for the reference strain, the candidate with the E340A mutation on NHMT, and the NHMT candidate carrying an mRFP-tag at the N-terminal or the C-terminal. **C)** Relative quantification as in panel C of the unmethylated counterpart peptide HTVIVYPGYR.

Supplementary Figure 4 – Multiple sequence alignment based prediction

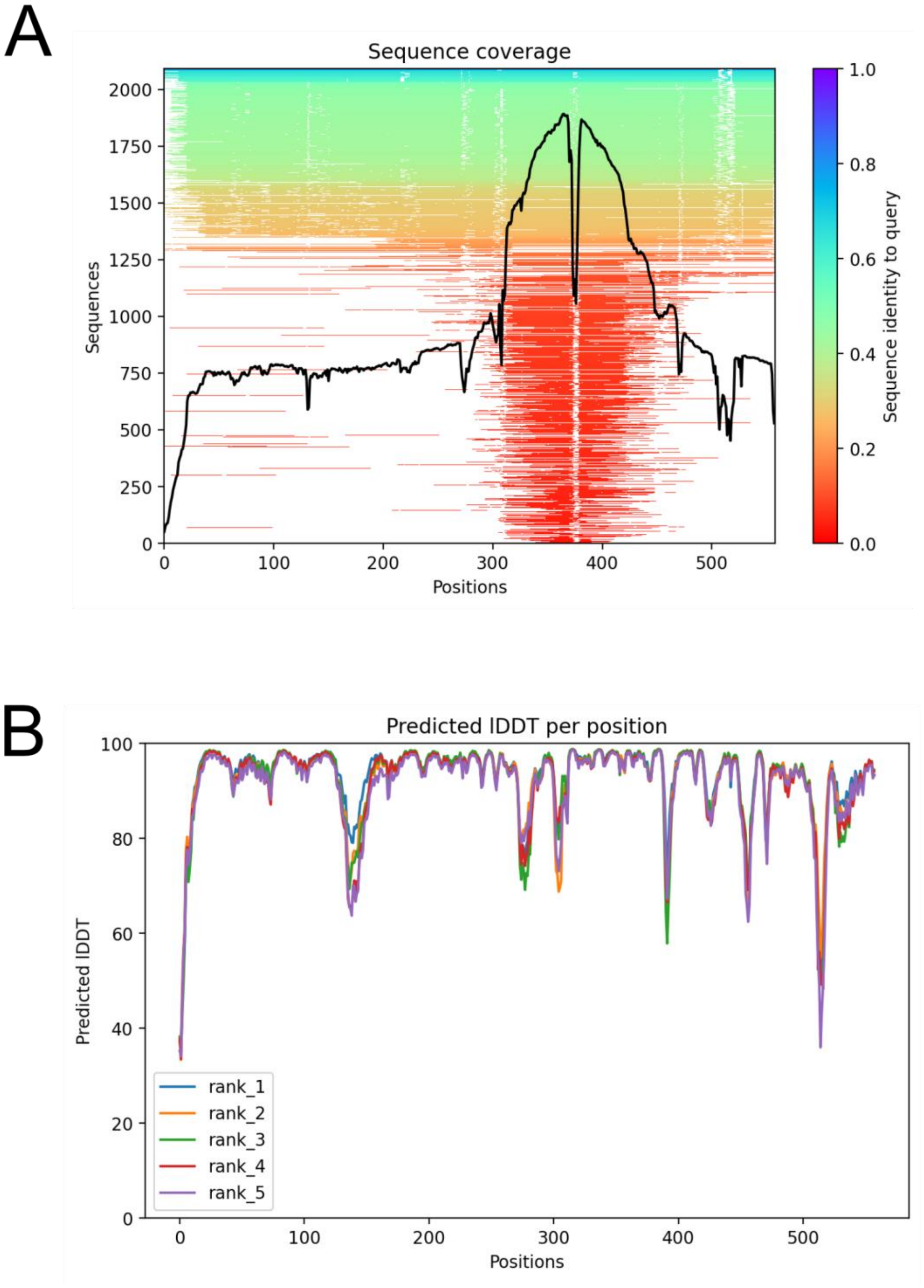

Supplementary Figure 4. A) Sequence coverage used in the multiple alignment that was based on the AlphaFold2 prediction. B) The prediction confidence which is an average local Distance Difference Test (lDDT) which is generally above 90%.

Supplementary Figure 5 – *In silico* analysis of the soluble domain

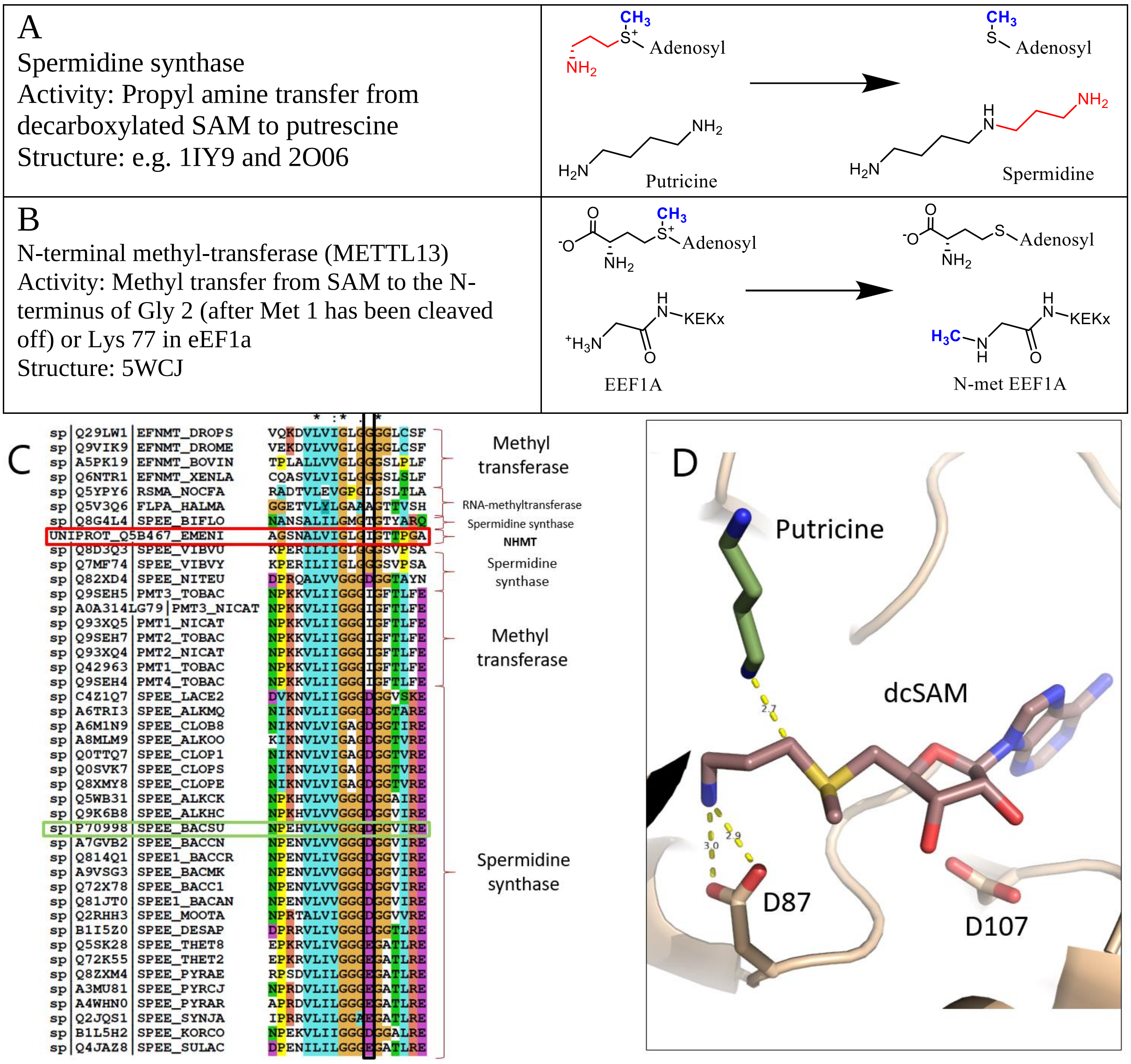

Supplementary Figure 5 – *In silico* analysis of the soluble domain. The substrate and co-substrates differ between **A)** spermidine synthases and **B)** methyl transferases. **C)** BLAST search of the soluble domain of NHMT (residues 216-558) against the manually curated database uniprotkb_swissprot, using the BLOSUM-45 matrix and an exp cutoff at 1.0 C). Framed in red, NHMT. Framed in green, the *B. subtilis* sequence that contains the spermidine synthase motif GxG(DE)G and is the most homologue sequence to NHMT where the experimental three-dimensional structure is available. The discriminating feature of spermidine synthases is the glycine rich loop where the acidic residue binds to the amine of decarboxylated SAM. **D)** The importance of the acidic residue is shown in an amalgam of structures from human (pdb:2O06), *T. cruzi* (pdb:4YV0) and *B. subtilis* (pdb:1IY9) spermidine synthases where the putrescine aligns perfectly for a nucleophilic attack on the decarboxylated SAM propylene moiety.

Supplementary Figure 6 – Sequence analysis of the 7TM domain shows that it is unique

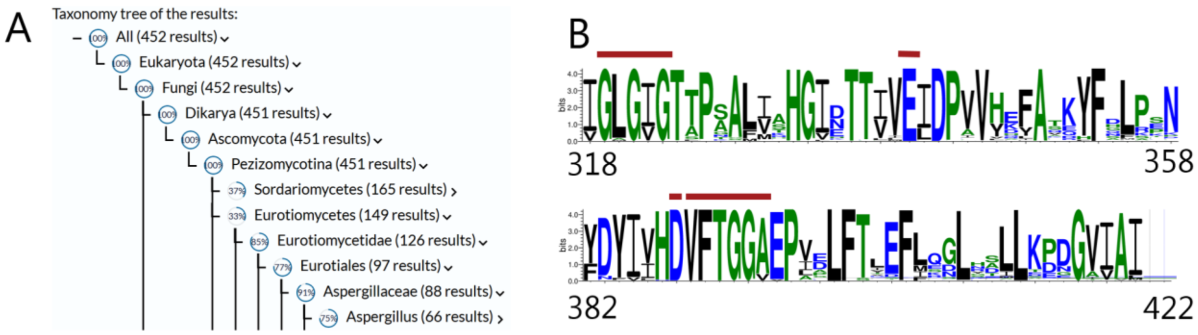

C

# No	Chain	Z	rmsd	%id	Description
1	6rw3-B	10.7	4.2	8	HEXOSE TRANSPORTER 1;
2	6kkk-A	10.3	4.3	9	SUGAR EFFLUX TRANSPORTER;
3	6g9x-A	10.3	3.8	10	MAJOR FACILITATOR SUPERFAMILY MFS_1;
4	4zp0-A	10.2	4.1	9	MULTIDRUG TRANSPORTER MDFA;
5	7bc6-A	9.9	4.1	11	PROTON-COUPLED FOLATE TRANSPORTER;
6	6t1z-A	9.7	4.5	9	LMRP INTEGRAL MEMBRANE PROTEIN;
7	7nqk-A	9.6	3.9	7	SOLUTE CARRIER FAMILY 15 MEMBER 2;
8	6s7v-A	9.5	4.1	13	MFS TRANSPORTER;
9	4u4t-A	9.3	4.3	11	NITRATE/NITRITE TRANSPORTER NARK;
10	2gfp-A	9.2	3.8	8	MULTIDRUG RESISTANCE PROTEIN D;
11	7d5p-B	9.2	3.9	9	DRUG TRANSPORTER, PUTATIVE;
12	7c76-B	9.1	4.6	7	TOLL-LIKE RECEPTOR 3;
13	6s4m-A	9.0	3.8	12	MAJOR FACILITATOR SUPERFAMILY ..
14	6gz9-A	9.0	4.2	12	PEPTIDE ABC TRANSPORTER PERMEASE;
15	6e9c-A	8.7	4.1	7	MAJOR FACILITATOR FAMILY TRANSPORTER;
16	3wdo-A	8.6	4.0	12	MFS TRANSPORTER;
17	6v4d-A	8.5	4.2	9	VESICULAR GLUTAMATE TRANSPORTER 2;
18	6e9n-A	8.4	4.0	8	D-GALACTONATE TRANSPORT;
19	1pw4-A	8.4	4.0	8	GLYCEROL-3-PHOSPHATE TRANSPORTER;
20	6wbv-A	8.0	4.3	9	HEPCIDIN;
21	3o7q-A	7.9	3.7	12	L-FUCOSE-PROTON SYMPORTER;
22	7dl9-A	7.8	4.0	8	NUCLEOSIDE PERMEASE NUPG;

Supplementary Figure 6 – Sequence analysis of the 7TM domain shows that it is unique. A) 452 sequences of homologous 7TM domains. All but one found in the Pezizomycotina phylum (filamentous ascomycetes). **B)** Alignment of the soluble domain linked to the 452 transmembrane domain sequences by MUSCLE v. 3.8 and visualized as sequence motifs in WebLogo 3.0. The region 318-358 shows the methyl transferase motif at 319-323 as well as the SAM binding glutamate in position 340. The highly conserved segment 389-394 is uniquely found in proteins with the nHMT 7TM domain. **C)** The first 22 hits found by search in the Dali25 database with the AlphaFold2 model of the An-nHMT 7TM domain. The structures of these proteins were investigated manually, none of which contain 7TM helices or domains.

Protein expression

Supplementary Figure 7 – LsAA9A secretion with the α-MF signal peptide

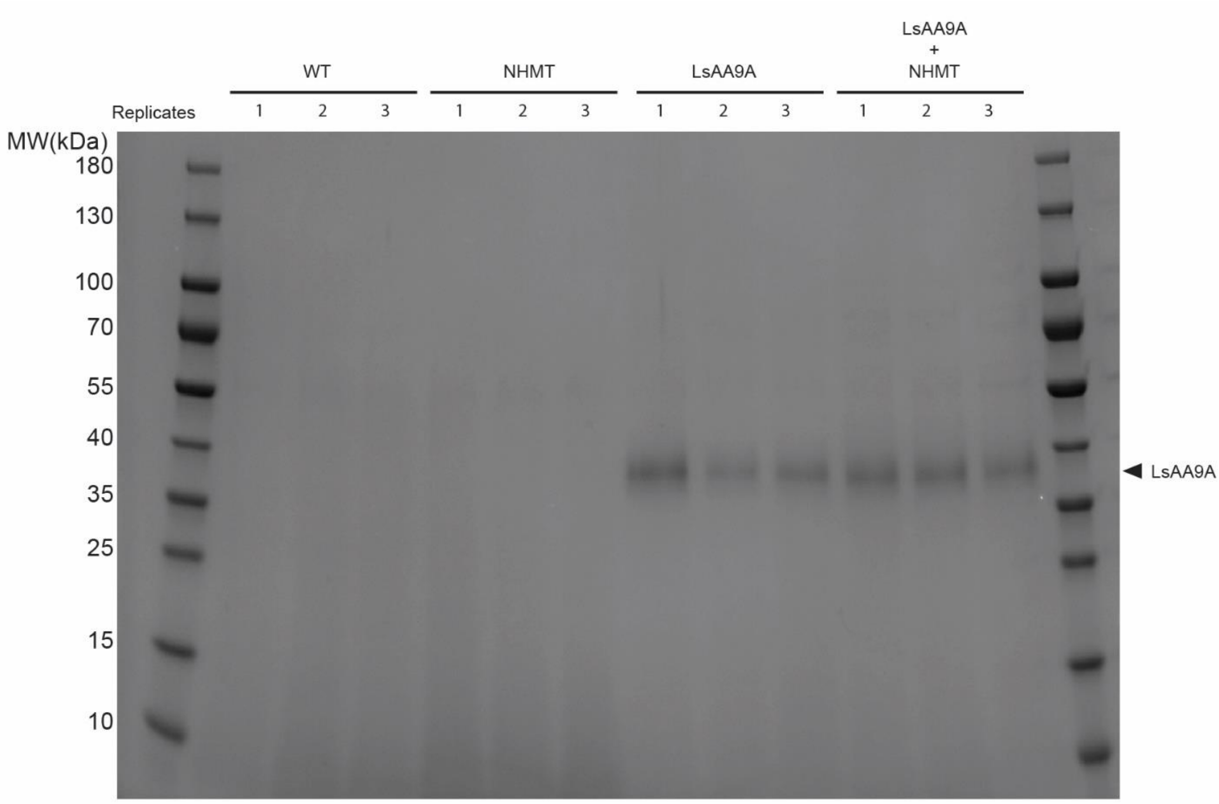

Supplementary Figure 7 – SDS-PAGE analysis of α-MF signal peptide-LsAA9A secretion in *K. phaffii*. MW, Marker in kDa. WT, GS115 parent strains. NHMT, strains carrying the episomal plasmid expressing the methyltransferase. LsAA9A, strains expressing LsAA9A with the α-MF signal peptide. LsAA9A+NHMT, strains co-expressing LsAA9A with the α-MF signal peptide and the methyltransferase. The molecular weight of LsAA9A is 25.2 kDa, howeverdue to glycosylation from the host *K. phaffii*, the secreted LsAA9A is found at around 37 kDa.

Supplementary Figure 8 – LsAA9A secretion with the Amy signal peptide

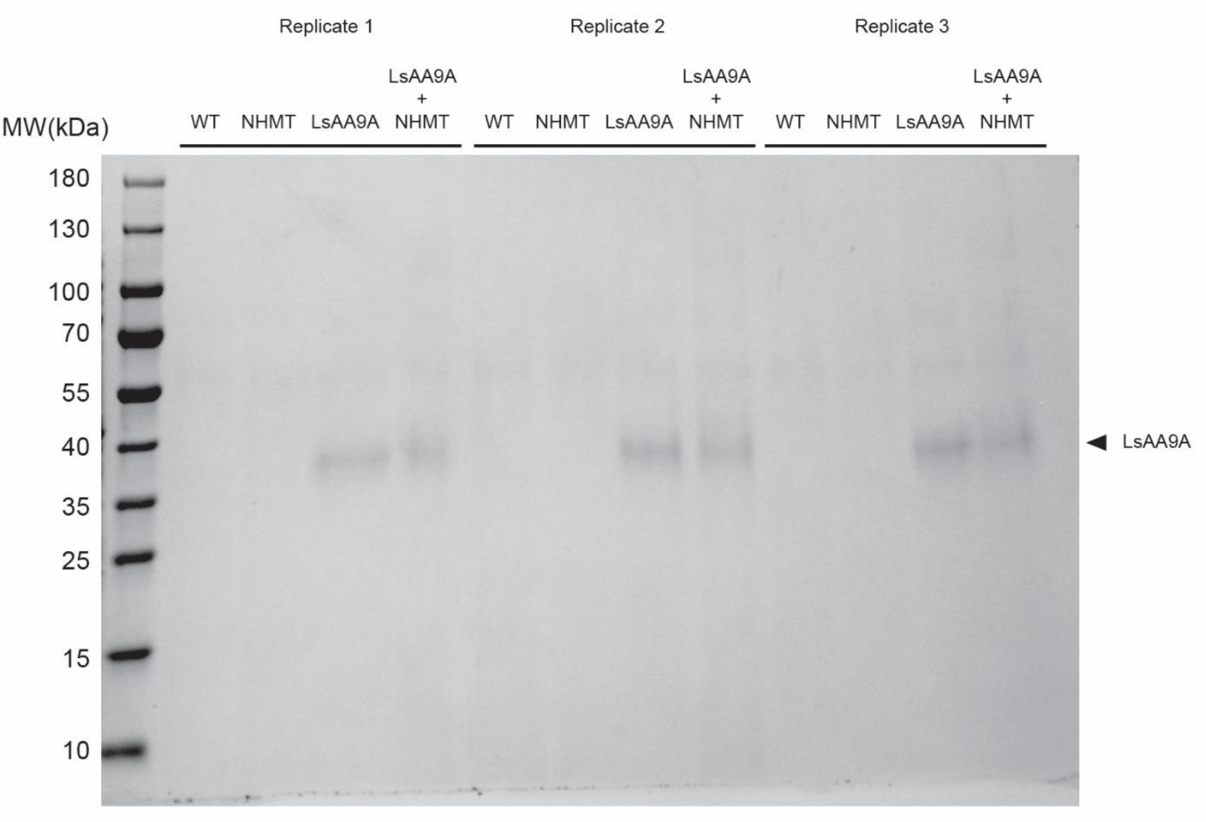

Supplementary Figure 8 – SDS-PAGE analysis of Amy signal peptide-LsAA9A secretion in *K. phaffii*. MW, Marker in kDa. WT, GS115 parent strains. NHMT, strains carrying the episomal plasmid expressing the methyltransferase. LsAA9A, strains expressing LsAA9A with the Amy signal peptide. LsAA9A+NHMT, strains co-expressing LsAA9A with the Amy signal peptide and the methyltransferase. The molecular weight of LsAA9A is 25.2 kDa. Due to glycosylation from the host *K. phaffii*, the secreted LsAA9A is found at around 37 kDa.

Supplementary Figure 9 – LsAA9A secretion with its native signal peptide

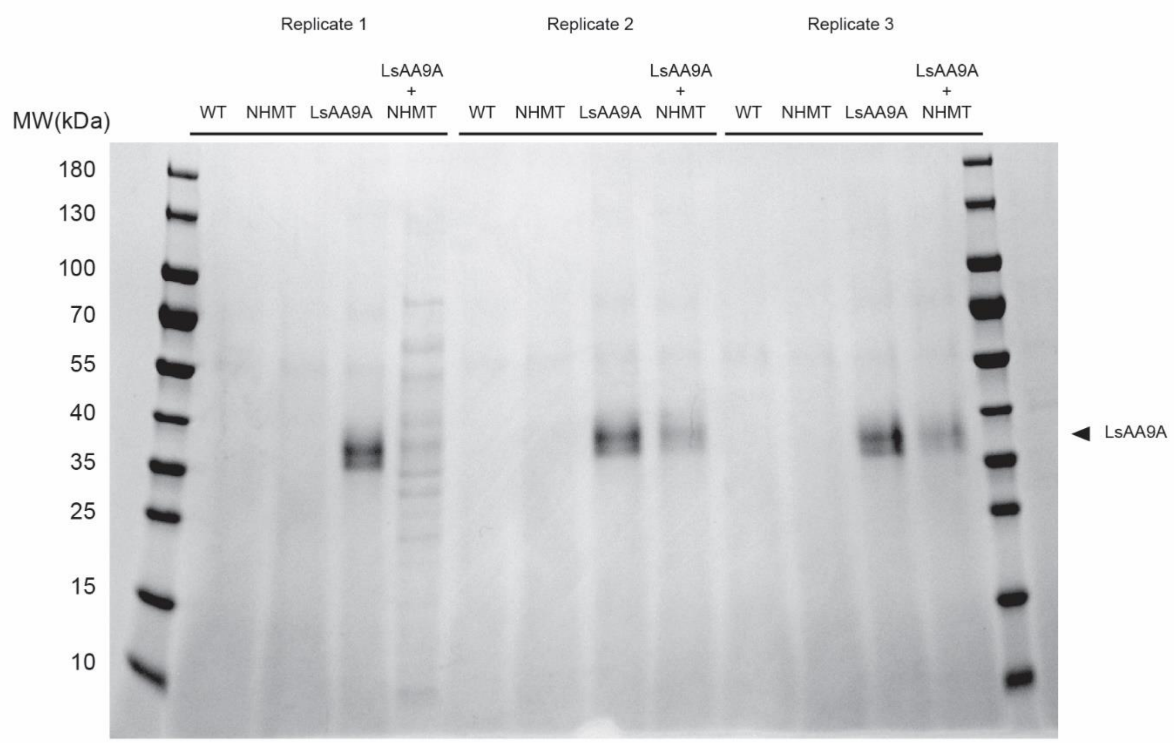

Supplementary Figure 9 – SDS-PAGE analysis of Native signal peptide LsAA9A secretion in *K. phaffii*. MW, Marker in kDa. WT, GS115 parent strains. NHMT, strains carrying the episomal plasmid expressing the methyltransferase. LsAA9A, strains expressing LsAA9A with the LsAA9A native signal peptide. LsAA9A+NHMT, strains co-expressing LsAA9A with the LsAA9A native signal peptide and the methyltransferase. The molecular weight of LsAA9A is 25.2 kDa. Due to glycosylation from the host *K. phaffii*, the secreted LsAA9A is found at around 37 kDa.

Supplementary Figure 10 – LsAA9A processing with alpha-mating factor signal peptide

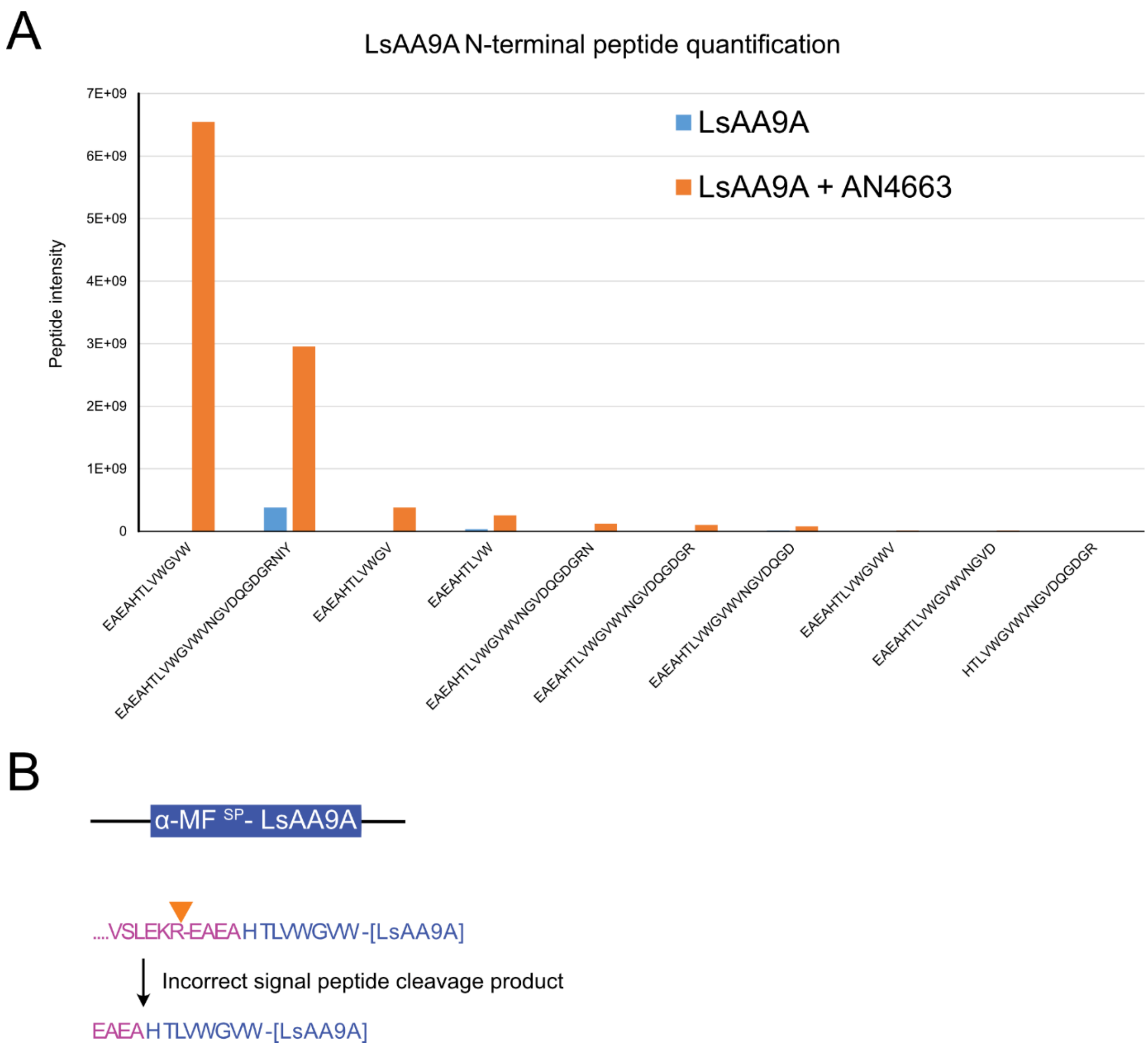

Supplementary Figure 10 – LsAA9A processing with alpha-mating factor signal peptide. LsAA9A N-terminal peptide MS identification and quantification with alpha-mating factor secretion signal peptide fused to the N-terminal of mature LsAA9A protein sequence. A) Quantification of identified N-terminal peptides of strains expressing LsAA9A (blue) with a alpha-mating factor signal peptide co-expressed with AN4663. B) Illustration of mature LsAA9A sequence with the addition of alpha-mating factor signal peptide.

Supplementary Figure 11 – TfAA10A secretion with the Amy signal peptide

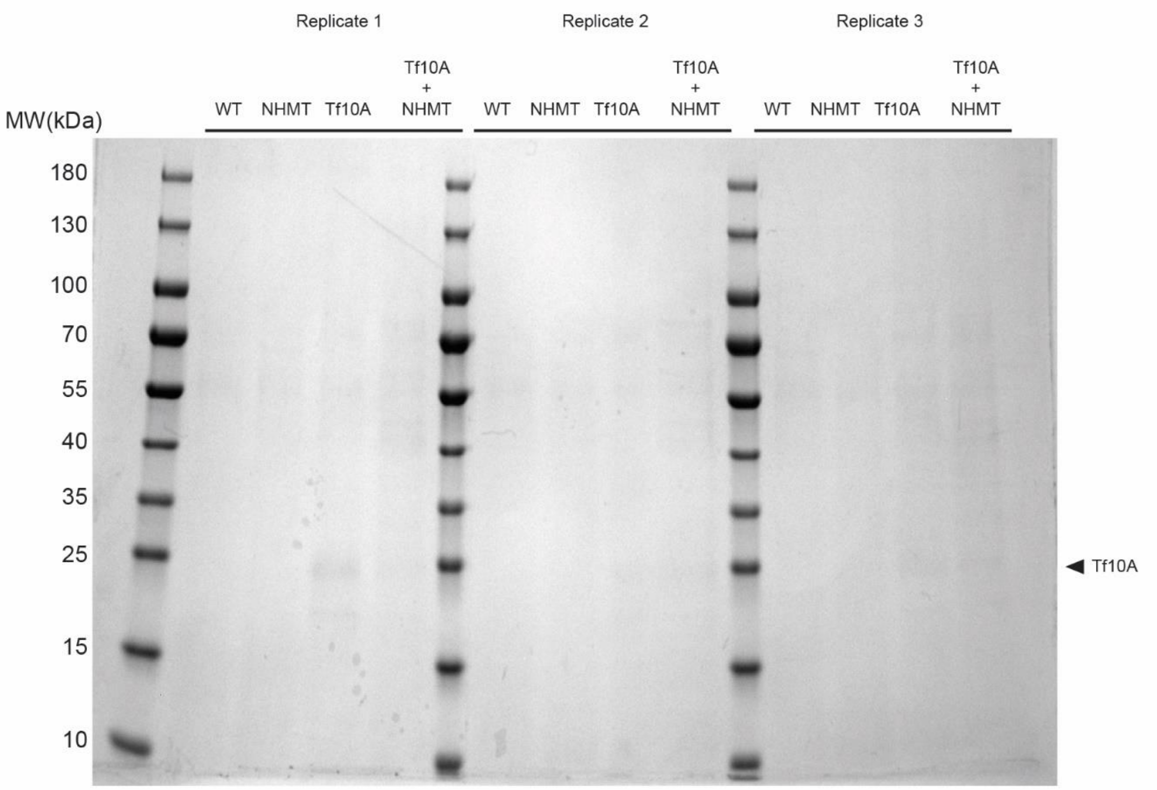

Supplementary Figure 11 – SDS-PAGE analysis of TfAA10A secretion in *K. phaffii*. MW, Marker in kDa. WT, GS115 parent strains. NHMT, strains carrying the episomal plasmid expressing the methyltransferase. Tf10A, strains expressing TfAA10A with the Amy signal peptide. Tf10A+NHMT, strains co-expressing TfAA10A with the Amy signal peptide and the methyltransferase. The molecular weight of TfAA10A is 21.3 kDa. Due to glycosylation from the host *K. phaffii*, the secreted TfAA10A is found at around 25 kDa.

Supplementary Table 1 – C8V530 pFam predication

Source	Domain	Start	End	Gathering threshold (bits)		Score (bits)		E-value	
				Sequence	Domain	Sequence	Domain	Sequence	Domain
**sig_p**	n/a	1	22	n/a	n/a	n/a	n/a	n/a	n/a
**low_complexity**	n/a	6	21	n/a	n/a	n/a	n/a	n/a	n/a
**Pfam**	AA9	23	242	27.8	27.8	226	225.7	1.50E-63	1.90E-63
**disorder**	n/a	50	53	n/a	n/a	n/a	n/a	n/a	n/a
**disorder**	n/a	58	59	n/a	n/a	n/a	n/a	n/a	n/a
**disorder**	n/a	63	64	n/a	n/a	n/a	n/a	n/a	n/a
**disorder**	n/a	76	79	n/a	n/a	n/a	n/a	n/a	n/a
**disorder**	n/a	216	221	n/a	n/a	n/a	n/a	n/a	n/a
**disorder**	n/a	267	271	n/a	n/a	n/a	n/a	n/a	n/a

Supplementary Table 1. This table includes pfam predicted domain of the *A. nidulans* protein C8V530 (Uniprot identifier). An auxiliary activity family 9 (AA9) domain with pfam accession PF03443 is predicted within C8V530.

Supplementary Table 2 - Q5B1W7 pFam predication.

Source	Domain	Start	End	Gathering threshold (bits)		Score (bits)		E-value	
				Sequence	Domain	Sequence	Domain	Sequence	Domain
**sig_p**	n/a	1	15	n/a	n/a	n/a	n/a	n/a	n/a
**Pfam**	LPMO_10	19	248	23	23	28.1	26.9	0.0092	0.022
**low_complexity**	n/a	252	281	n/a	n/a	n/a	n/a	n/a	n/a
**disorder**	n/a	259	270	n/a	n/a	n/a	n/a	n/a	n/a
**Pfam**	CBM_20	283	379	23.3	23.3	121	120.2	4.80E-32	8.40E-32
**low_complexity**	n/a	315	329	n/a	n/a	n/a	n/a	n/a	n/a
**disorder**	n/a	360	361	n/a	n/a	n/a	n/a	n/a	n/a
**disorder**	n/a	369	372	n/a	n/a	n/a	n/a	n/a	n/a
**disorder**	n/a	384	385	n/a	n/a	n/a	n/a	n/a	n/a

Supplementary Table 2. This table includes pfam predicted domain of the *A. nidulans* protein Q5B1W7 (Uniprot identifier). A conserved lytic polysaccharide monooxygenase, cellulose-degrading domain (LPMO_10) as well as a carbohydrate/starch binding domain (CBM_20) with pfam accessions PF03067 and PF00686 are respectively predicted within Q5B1W7. The protein is considered part of the AA13 family of LPMOs by CAZy.

Supplementary Table 3 - *Aspergillus nidulans* strains used in this study.

**Strain ID**	**Modified site/s**[1]	**Genotype**	**Reference**
NID174/FGSC A4	-	-	NCBI:txid227321
NID2531	Host/reference	*argB2, veA1, nkuA*Δ	In house collection
NID2134	IS1[2]	*argB2; pyrG89; veA1; nkuA*Δ; *IS1::*P*gpdA-AN10118-RFP*-T*trpC::argB; IS4::*P*gpdA-mpaA-mCitrine*-T*trpC::AFpyrG*	In house collection
NID2838	AN10909	*argB2, veA1, nkuA*Δ, AN10909Δ	This study
NID2714	AN2165	*argB2, veA1, nkuA*Δ, AN2165Δ	This study
NID2721	AN1566	*argB2, veA1, nkuA*Δ, AN1566Δ	This study
NID2715	AN0761	*argB2, veA1, nkuA*Δ, AN0761Δ	This study
NID2733	AN0134	*argB2, veA1, nkuA*Δ, AN0134Δ	This study
NID2709	AN2405	*argB2, veA1, nkuA*Δ, AN2405Δ	This study
NID2713	AN4663	*argB2, veA1, nkuA*Δ, AN4663Δ	This study
NID2773	AN4663	*argB2, veA1, nkuA*Δ, AN4663Δ	This study
NID2774	AN4663	*argB2, veA1, nkuA*Δ, AN4663Δ	This study
NID2775	AN4663	*argB2, veA1, nkuA*Δ, AN4663Δ	This study
NID2723	AN7375	*argB2, veA1, nkuA*Δ, AN7375Δ	This study
NID2712	AN10974	*argB2, veA1, nkuA*Δ, AN10974Δ	This study
NID2745	AN8945	*argB2, veA1, nkuA*Δ, AN8945Δ	This study
NID2746	AN9193	*argB2, veA1, nkuA*Δ, AN9193Δ	This study
NID2747	AN4625	*argB2, veA1, nkuA*Δ, AN4625Δ	This study
NID2749	AN5874	*argB2, veA1, nkuA*Δ, AN5874Δ	This study
NID2750	AN10700	*argB2, veA1, nkuA*Δ, AN10700Δ	This study
NID2755	AN5630	*argB2, veA1, nkuA*Δ, AN5630Δ	This study
NID2757	AN2406	*argB2, veA1, nkuA*Δ, AN2406Δ	This study
NID2759	AN9098	*argB2, veA1, nkuA*Δ, AN9098Δ	This study
NID2802	AN6094	*argB2, veA1, nkuA*Δ, AN6094Δ	This study
NID2801	AN3096	*argB2, veA1, nkuA*Δ, AN3096Δ	This study

NID2761	AN8849	*argB2*, *veA1*, *nkuA*Δ, AN8849Δ	This study
NID2710	AN3464	*argB2*, *veA1*, *nkuA*Δ, AN3464Δ	This study
NID2825	AN6713	*argB2*, *veA1*, *nkuA*Δ, AN6713Δ	This study
NID2756	AN4663 & IS5	*argB2*, *veA1*, *nkuA*Δ, AN4663Δ, IS5::Ptef-AN4663-Ttef::	This study
NID2751	AN4663 & IS5	*argB2*, *veA1*, *nkuA*Δ, IS5::Ptef-AN4663-Ttef::	This study
NID2787	AN4663	*argB2*, *veA1*, *nkuA*Δ, AN4663::E340A::	This study
NID2815	AN4663	*argB2*, *veA1*, *nkuA*Δ, AN4663::E340A::	This study
NID2789	AN4663 & IS5	*argB2*, *veA1*, *nkuA*Δ, AN4663Δ, IS5::Ptef-*mRFP*-AN4663-Ttef::	This study
NID2794	AN4663 & IS5	*argB2*, *veA1*, *nkuA*Δ, AN4663Δ, IS5::Ptef-AN4663-*mRFP*-Ttef::	This study
NID2858	AN4663 & IS5	*argB2*, *veA1*, *nkuA*Δ, AN4663Δ, IS5::Pan4663-AN4663-Tan4663::	This study
NID2851	AN4663 & IS5	argB2, veA1, nkuAΔ, AN4663Δ, IS5::Pan4663-*mRFP*-AN4663-Tan4663::	This study
NID2852	AN4663 & IS5	*argB2*, *veA1*, *nkuA*Δ, AN4663Δ, IS5::Pan4663-*mRFP*-AN4663-Tan4663::	This study
NID2853	AN4663 & IS5	*argB2*, *veA1*, *nkuA*Δ, AN4663Δ, IS5::Pan4663-*mRFP*-AN4663-Tan4663::	This study
NID2843	AN4663 & IS5	*argB2*, *veA1*, *nkuA*Δ, AN4663Δ, IS5::Pan4663-Truncated_AN4663-Tan4663::	This study
NID2844	AN4663 & IS5	*argB2*, *veA1*, *nkuA*Δ, AN4663Δ, IS5::Pan4663-Truncated_AN4663-Tan4663::	This study
NID2845	AN4663 & IS5	*argB2*, *veA1*, *nkuA*Δ, AN4663Δ, IS5::Pan4663-Truncated_AN4663-Tan4663::	This study
NID2857	IS5	*argB2*, *veA1*, *nkuA*Δ, IS5::Ptef-*mRFP*-Ttef::	This Study

[1] Genomic loci altered compared with NID2531

[2] IS1 for integration site 1 from Hansen et al, 2011 (Appl. Environ. Microbiol., 77 [2011]) ; IS5 is from from Holm et al, 2013 (PhD Thesis: Development and Implementation of Novel Genetic Tools for Investigation of Fungal Secondary Metabolism. Technical University of Denmark.), and Schalén et al., 2016 (Fungal Biol. Biotechnol. 3, 3).

Supplementary Table 4 - *K. phaffii* strains used in this study.

**Strain**	**Genotype**	**Source/Reference**
E. coli NEB5α	*fhuA2* Δ(*argF-lacZ*)U169 *phoA glnV44* Φ80 Δ(*lacZ*)M15 *gyrA96 recA1 relA1 endA1 thi-1 hsdR17*	a
K. phaffii (P. pastoris) GS115	*his4*	b
K. phaffii GS115 α-MF^{SP}-LsAA9A Mut^{+}	*aox1*::P_{AOX1}-α-MF^{SP}-LsAA9A-His6-*HIS4*	This study
K. phaffii GS115 Amy^{SP}-LsAA9A Mut^{+}	*aox1*::P_{AOX1}-Amy^{SP}-LsAA9A-His6-*HIS4*	This study
K. phaffii GS115 Native^{SP}-LsAA9A Mut^{+}	*aox1*::P_{AOX1}-Native^{SP}-LsAA9A-His6-*HIS4*	This study
K. phaffii GS115 α-MF^{SP}-LsAA9A Mut^{+}	*aox1*::P_{AOX1}-α-MF^{SP}-LsAA9A-His6-*HIS4* + pBGP1-AN4663	This study
K. phaffii GS115 Amy^{SP}-LsAA9A Mut^{+}	*aox1*::P_{AOX1}-Amy^{SP}-LsAA9A-His6-*HIS4* + pBGP1-AN4663	This study
K. phaffii GS115 Native^{SP}-LsAA9A Mut^{+}	*aox1*::P_{AOX1}-Native^{SP}-LsAA9A-His6-*HIS4* + pBGP1-AN4663	This study
K. phaffii GS115 Amy^{SP}-Tf10A Mut^{+}	*aox1*::P_{AOX1}-Amy^{SP}-Tf10A-*HIS4*	This study
K. phaffii GS115 Amy^{SP}-Tf10A Mut^{+}	*aox1*::P_{AOX1}-Amy^{SP}-Tf10A-*HIS4* + pBGP1-AN4663	This study

[a] NEB, Ipswich, MA, USA; [b]Thermo Fisher Scientific, Waltham, MA, USA

Supplementary Table 5 - Plasmids used in this study.

**Plasmid**	**Description**	**Reference**
pLyGo-*Kp*-1	Integrative vector into the AOX1 locus. Encodes the α-MF signal peptide for secretion. Km^R (*E. coli*), *HIS4* (*K. phaffii*)	[1]
pLyGo-Kp-1-LsAA9A	Integrative vector into the AOX1 locus. Encodes the α-MF signal peptide for secretion and LsAA9A. Km^R (*E. coli*), *HIS4* (*K. phaffii*)	[1]
pLyGo-*Kp*-2	Integrative vector into the AOX1 locus. Encodes the Amy signal peptide for secretion. Km^R (*E. coli*), *HIS4* (*K. phaffii*)	[1]
pLyGo-*Kp*-2-LsAA9A	Integrative vector into the AOX1 locus. Encodes the Amy signal peptide for secretion and LsAA9A. Km^R (*E. coli*), *HIS4* (*K. phaffii*)	1
pLyGo-*Kp*-2-Tf10A	Integrative vector into the AOX1 locus. Encodes the Amy signal peptide for secretion and Tf10A. Km^R (*E. coli*), *HIS4* (*K. phaffii*)	This study
pPIC9K-$Native^{SP}$-LsAA9A	Integrative vector into the AOX1 locus. Encodes the Native signal peptide for secretion of LsAA9A and LsAA9A. Km^R (*E. coli*), *HIS4* (*K. phaffii*)	This study
pBGP1	Episomal vector with a pGAP constitute promoter, Amp^R (*E. coli*), $Zeocin^R$ (*K. phaffii*)	[2]
pFC331	CRISPR-Cas9 vector, Amp^R (*E. coli*), ORI, *argB* (*A. nidulans)*, AMA1.	[3]
pcAN10909	CRISPR-Cas9 vector, Amp^R (*E. coli*), ORI, *argB* (*A. nidulans)*, AMA1, sgRNA targeting AN10909 flanked by self-splicing tRNA.	This study
pcAN2165	CRISPR-Cas9 vector, Amp^R (*E. coli*), ORI, *argB* (*A. nidulans)*, AMA1, sgRNA targeting AN2165 flanked by self-splicing tRNA.	This study
pcAN1566	CRISPR-Cas9 vector, Amp^R (*E. coli*), ORI, *argB* (*A. nidulans)*, AMA1, sgRNA targeting AN1566 flanked by self-splicing tRNA.	This study
pcAN0761	CRISPR-Cas9 vector, Amp^R (*E. coli*), ORI, *argB* (*A. nidulans)*, AMA1, sgRNA targeting AN0761 flanked by self-splicing tRNA.	This study
pcAN0134	CRISPR-Cas9 vector, Amp^R (*E. coli*), ORI, *argB* (*A. nidulans)*, AMA1, sgRNA targeting AN0134 flanked by self-splicing tRNA.	This study
pcAN2405	CRISPR-Cas9 vector, Amp^R (*E. coli*), ORI, *argB* (*A. nidulans)*, AMA1, sgRNA targeting AN2405 flanked by self-splicing tRNA.	This study

pcAN4663	CRISPR-Cas9 vector, Amp^R (*E. coli*), ORI, *argB* (*A. nidulans)*, AMA1, sgRNA targeting AN4663 flanked by self-splicing tRNA.	This study
pcAN7375	CRISPR-Cas9 vector, Amp^R (*E. coli*), ORI, *argB* (*A. nidulans)*, AMA1, sgRNA targeting AN7375 flanked by self-splicing tRNA.	This study
pcAN10974	CRISPR-Cas9 vector, Amp^R (*E. coli*), ORI, *argB* (*A. nidulans)*, AMA1, sgRNA targeting AN10974 flanked by self-splicing tRNA.	This study
pcAN8945	CRISPR-Cas9 vector, Amp^R (*E. coli*), ORI, *argB* (*A. nidulans)*, AMA1, sgRNA targeting AN8945 flanked by self-splicing tRNA.	This study
pcAN9193	CRISPR-Cas9 vector, Amp^R (*E. coli*), ORI, *argB* (*A. nidulans)*, AMA1, sgRNA targeting AN9193 flanked by self-splicing tRNA.	This study
pcAN3464	CRISPR-Cas9 vector, Amp^R (*E. coli*), ORI, *argB* (*A. nidulans)*, AMA1, sgRNA targeting AN3464 flanked by self-splicing tRNA.	This study
pcAN4625	CRISPR-Cas9 vector, Amp^R (*E. coli*), ORI, *argB* (*A. nidulans)*, AMA1, sgRNA targeting AN4625 flanked by self-splicing tRNA.	This study
pcAN6094	CRISPR-Cas9 vector, Amp^R (*E. coli*), ORI, *argB* (*A. nidulans)*, AMA1, sgRNA targeting AN6094 flanked by self-splicing tRNA.	This study
pcAN9098	CRISPR-Cas9 vector, Amp^R (*E. coli*), ORI, *argB* (*A. nidulans)*, AMA1, sgRNA targeting AN9098 flanked by self-splicing tRNA.	This study
pcAN3096	CRISPR-Cas9 vector, Amp^R (*E. coli*), ORI, *argB* (*A. nidulans)*, AMA1, sgRNA targeting AN3096 flanked by self-splicing tRNA.	This study
pcAN5874	CRISPR-Cas9 vector, Amp^R (*E. coli*), ORI, *argB* (*A. nidulans)*, AMA1, sgRNA targeting AN5874 flanked by self-splicing tRNA.	This study
pcAN6713	CRISPR-Cas9 vector, Amp^R (*E. coli*), ORI, *argB* (*A. nidulans)*, AMA1, sgRNA targeting AN6713 flanked by self-splicing tRNA.	This study
pcAN8849	CRISPR-Cas9 vector, Amp^R (*E. coli*), ORI, *argB* (*A. nidulans)*, AMA1, sgRNA targeting AN8849 flanked by self-splicing tRNA.	This study
pcAN10700	CRISPR-Cas9 vector, Amp^R (*E. coli*), ORI, *argB* (*A. nidulans)*, AMA1, sgRNA targeting AN10700 flanked by self-splicing tRNA.	This study
pcAN5630	CRISPR-Cas9 vector, Amp^R (*E. coli*), ORI, *argB* (*A. nidulans)*, AMA1, sgRNA targeting AN5630 flanked by self-splicing tRNA.	This study
pcAN2406	CRISPR-Cas9 vector, Amp^R (*E. coli*), ORI, *argB* (*A. nidulans)*, AMA1, sgRNA targeting AN2406 flanked by self-splicing tRNA.	This study

pcIS5	CRISPR-Cas9 vector, Amp^R (*E. coli*), ORI, *argB* (*A. nidulans)*, AMA1, sgRNA targeting IS5 flanked by self-splicing tRNA.	in house collection
pcAN4663-PM-PS#2	CRISPR-Cas9 vector, Amp^R (*E. coli*), ORI, *argB* (*A. nidulans)*, AMA1, sgRNA targeting E340 of AN4663 flanked by self-splicing tRNA.	This study
pIS5-AN4663	Amp^R (*E. coli*), ORI. Integration vector for insertion of Ptef, AN4663, and Ttef in integration site 5	This study
pIS5-AN4663-N-tag-mRFP	Amp^R (*E. coli*), ORI. Integration vector for insertion of AN4663 with Ptef promoter and Ttef terminator and N-terminal tagged with mRFP in integration site 5	This study
pIS5-AN4663-C-tag-mRFP	Amp^R (*E. coli*), ORI. Integration vector for insertion of AN4663 with Ptef promoter and Ttef terminator and C-terminal tagged with mRFP in integration site 5	This study
pIS5-Pan4663-AN4663-Tan4663	Amp^R (*E. coli*), ORI. Integration vector for insertion of AN4663 with native promoter and terminator in integration site 5	This study
pIS5-AN4663-TruncV2 Native	Amp^R (*E. coli*), ORI. Integration vector for insertion of truncated AN4663 after membrane region with native promoter and terminator in integration site 5	This study
pIS5-Ptef-mRFP-Ttef	Amp^R (*E. coli*), ORI. Integration vector for insertion of Ptef, mRFP, and Ttef in integration site 5	This study

Supplementary Table 6 – Oligonucleotides and target sequences used in this study.

Color code for: annealing sequence, protospacer sequence, thymine → uracil substitution, overhangs for PacI/Nt.BbvCI cassette

ID	Purpose	Sequence (5’ à 3’)
**Primers CRISPR/Cas9-vector construction**		
CSN438-Afum-U3p-fwd	Amplifying CRISPR fragment with promoter	GGGTTTAAUGATCACATAGATGCTCGGTTGACA
CSN790-U3-term-rv	Amplifying CRISPR fragment with terminator	GGTCTTAAUACCCTGAGAAGATAGATGTGAATGTG
tRNA-gRNA328-rv	Amplifying CRISPR fragment with PS tails targeting AN10909	AGGACATTAUGCACTCGTGCATCATCCGTGAATCGAAC
gRNA328-fwd	Amplifying CRISPR fragment with PS tails targeting AN10909	ATAATGTCCUTCAGTTTTAGAGCTAGAAATAGCAAGTTAAA
tRNA-gRNA329-rv	Amplifying CRISPR fragment with PS tails targeting AN10909	ATGCAGGTGGUGGGTGCATCATCCGTGAATCGAAC
gRNA329-fwd	Amplifying CRISPR fragment with PS tails targeting AN10909	ACCACCTGCAUTAGTTCGTTTTAGAGCTAGAAATAGCAAGTTAAA
tRNA-gRNA330-rv	Amplifying CRISPR fragment with PS tails targeting AN2165	ATCTGCTCATGGAAAUTTTGCATCATCCGTGAATCGAAC
gRNA330-fwd	Amplifying CRISPR fragment with PS tails targeting AN2165	ATTTCCATGAGCAGAUTCGTTTTAGAGCTAGAAATAGCAAGTTAAA
tRNA-gRNA331-rv	Amplifying CRISPR fragment with PS tails targeting AN2165	AGTTCATTGCTUCGCGTGCATCATCCGTGAATCGAAC
gRNA331-fwd	Amplifying CRISPR fragment with PS tails targeting AN2165	AAGCAATGAACUGCTCGTTTTAGAGCTAGAAATAGCAAGTTAAA
tRNA-gRNA332-rv	Amplifying CRISPR fragment with PS tails targeting AN1566	ACCAGTTGTCCUTATGCATCATCCGTGAATCGAAC
gRNA332-fwd	Amplifying CRISPR fragment with PS tails targeting AN1566	AGGACAACTGGUCATCCGGTTTTAGAGCTAGAAATAGCAAGTTAAA
tRNA-gRNA333-rv	Amplifying CRISPR fragment with PS tails targeting AN1566	AACATACCCUAGCCTGCATCATCCGTGAATCGAAC
gRNA333-fwd	Amplifying CRISPR fragment with PS tails targeting AN1566	AGGGTATGTUAGGCTTGTTTTAGAGCTAGAAATAGCAAGTTAAA
tRNA-gRNA338-rv	Amplifying CRISPR fragment with PS tails targeting AN0761	ATCTCAGCACCUCCCGATTGCATCATCCGTGAATCGAAC
gRNA338-fwd	Amplifying CRISPR fragment with PS tails targeting AN0761	AGGTGCTGAGAUGAGTTTTAGAGCTAGAAATAGCAAGTTAAA
tRNA-gRNA339-rv	Amplifying CRISPR fragment with PS tails targeting AN0761	AGGGCAGAAGGGUGATGCATCATCCGTGAATCGAAC
gRNA339-fwd	Amplifying CRISPR fragment with PS tails targeting AN0761	ACCCTTCTGCCCUATTTGGTTTTAGAGCTAGAAATAGCAAGTTAAA
tRNA-gRNA340-rv	Amplifying CRISPR fragment with PS tails targeting AN0134	AGATTCCAUACTGTGCATCATCCGTGAATCGAAC
gRNA340-fwd	Amplifying CRISPR fragment with PS tails targeting AN0134	ATGGAATCUGAAAGCGGTTTTAGAGCTAGAAATAGCAAGTTAAA
tRNA-gRNA341-rv	Amplifying CRISPR fragment with PS tails targeting AN0134	AATGCTTGAGUGTATGCATCATCCGTGAATCGAAC
gRNA341-fwd	Amplifying CRISPR fragment with PS tails targeting AN0134	ACTCAAGCATUAAGGATGTTTTAGAGCTAGAAATAGCAAGTTAAA
tRNA-gRNA-AN4663-rv(B)	Amplifying CRISPR fragment with PS tails targeting AN4663	ACCAGCTTCTTGGUAGCTGCATCATCCGTGAATCGAAC
gRNA_AN4663-fwd(B)	Amplifying CRISPR fragment with PS tails targeting AN4663	ACCAAGAAGCTGGUAGTGTTTTAGAGCTAGAAATAGCAAGTTAAA
tRNA-gRNA-AN4663-rv(E)	Amplifying CRISPR fragment with PS tails targeting AN4663	ACAGTCCTCAUAATCTGCATCATCCGTGAATCGAAC
gRNA_AN4663-fwd(E)	Amplifying CRISPR fragment with PS tails targeting AN4663	ATGAGGACTGUTTTGCGTTTTAGAGCTAGAAATAGCAAGTTAAA
tRNA-gRNA-AN3464-rv(B)	Amplifying CRISPR fragment with PS tails targeting AN3464	AGTACCACGCCAUCGTTGCATCATCCGTGAATCGAAC
gRNA_AN3464-fwd(B)	Amplifying CRISPR fragment with PS tails targeting AN3464	ATGGCGTGGTACUGTTCGTTTTAGAGCTAGAAATAGCAAGTTAAA

tRNA-gRNA-AN3464-rv(E)	Amplifying CRISPR fragment with PS tails targeting AN3464	AATGCGGTACUGATGCATCATCCGTGAATCGAAC
gRNA_AN3464-fwd(E)	Amplifying CRISPR fragment with PS tails targeting AN3464	AGTACCGCATUCGGCGGTGTTTTAGAGCTAGAAATAGCAAGTTAAA
tRNA-gRNA-AN2405-rv(B)	Amplifying CRISPR fragment with PS tails targeting AN2405	ATTGCTTTTGCUCTGCATCATCCGTGAATCGAAC
gRNA_AN2405-fwd(B)	Amplifying CRISPR fragment with PS tails targeting AN2405	AGCAAAAGCAAUCCACCTTGTTTTAGAGCTAGAAATAGCAAGTTAAA
tRNA-gRNA-AN2405-rv(E)	Amplifying CRISPR fragment with PS tails targeting AN2405	ACCTTCTTTGUCAACGTCTGCATCATCCGTGAATCGAAC
gRNA_AN2405-fwd(E)	Amplifying CRISPR fragment with PS tails targeting AN2405	ACAAAGAAGGUCGGTTTTAGAGCTAGAAATAGCAAGTTAAA
tRNA-gRNA-AN7375-rv(B)	Amplifying CRISPR fragment with PS tails targeting AN7375	AGCTCGACTGAUTTCTTTGCATCATCCGTGAATCGAAC
gRNA_AN7375-fwd(B)	Amplifying CRISPR fragment with PS tails targeting AN7375	ATCAGTCGAGCUCTCGTTTTAGAGCTAGAAATAGCAAGTTAAA
tRNA-gRNA-AN7375-rv(E)	Amplifying CRISPR fragment with PS tails targeting AN7375	AGGCAACACCGUTTGCATCATCCGTGAATCGAAC
gRNA_AN7375-fwd(E)	Amplifying CRISPR fragment with PS tails targeting AN7375	ACGGTGTTGCCUCTCAGTTGTTTTAGAGCTAGAAATAGCAAGTTAAA
tRNA-gRNA-AN10974-rv(B)	Amplifying CRISPR fragment with PS tails targeting AN10974	ATGTCGACTATGUCAATGCATCATCCGTGAATCGAAC
gRNA_AN10974-fwd(B)	Amplifying CRISPR fragment with PS tails targeting AN10974	ACATAGTCGACAUTCGCGTTTTAGAGCTAGAAATAGCAAGTTAAA
tRNA-gRNA-AN10974-rv(E)	Amplifying CRISPR fragment with PS tails targeting AN10974	ACCGCTTCCAAACUCATGCATCATCCGTGAATCGAAC
gRNA_AN10974-fwd(E)	Amplifying CRISPR fragment with PS tails targeting AN10974	AGTTTGGAAGCGGUTCCGGTTTTAGAGCTAGAAATAGCAAGTTAAA
tRNA-gRNA-AN9193-rv1	Amplifying CRISPR fragment with PS tails targeting AN9193	ATGGCTGACGCUGCATGCATCATCCGTGAATCGAAC
gRNA-AN9193-fwd1	Amplifying CRISPR fragment with PS tails targeting AN9193	AGCGTCAGCCAUGGTCAGTTTTAGAGCTAGAAATAGCAAGTTAAA
tRNA-gRNA-AN9193-rv2	Amplifying CRISPR fragment with PS tails targeting AN9193	ACAACATTGAACCUGCTGCATCATCCGTGAATCGAAC
gRNA-AN9193-fwd2	Amplifying CRISPR fragment with PS tails targeting AN9193	AGGTTCAATGTTGUGGTCGTTTTAGAGCTAGAAATAGCAAGTTAAA
tRNA-gRNA-AN8945-rv1	Amplifying CRISPR fragment with PS tails targeting AN8945	ACCGACAAAAUATGCATCATCCGTGAATCGAAC
gRNA-AN8945-fwd1	Amplifying CRISPR fragment with PS tails targeting AN8945	ATTTTGTCGGUCGCAATGGGTTTTAGAGCTAGAAATAGCAAGTTAAA
tRNA-gRNA-AN8945-rv2	Amplifying CRISPR fragment with PS tails targeting AN8945	ACTCCGGCTTCUGCCTGCATCATCCGTGAATCGAAC
gRNA-AN8945-fwd2	Amplifying CRISPR fragment with PS tails targeting AN8945	AGAAGCCGGAGUAGAGCGTTTTAGAGCTAGAAATAGCAAGTTAAA
tRNA-gRNA-AN4625-rv1	Amplifying CRISPR fragment with PS tails targeting AN4625	AGTAGGTGTCTUGAATCTGCATCATCCGTGAATCGAAC
gRNA-AN4625-fwd1	Amplifying CRISPR fragment with PS tails targeting AN4625	AAGACACCTACUCCGGTTTTAGAGCTAGAAATAGCAAGTTAAA
tRNA-gRNA-AN4625-rv2	Amplifying CRISPR fragment with PS tails targeting AN4625	ACAGCATAGAUCTGTGCATCATCCGTGAATCGAAC
gRNA-AN4625-fwd2	Amplifying CRISPR fragment with PS tails targeting AN4625	ATCTATGCTGUCAAGGGGTTTTAGAGCTAGAAATAGCAAGTTAAA
tRNA-gRNA-AN6094-rv1	Amplifying CRISPR fragment with PS tails targeting AN6094	ATGGGTTGTUGTGAAGGTGCATCATCCGTGAATCGAAC
gRNA-AN6094-fwd1	Amplifying CRISPR fragment with PS tails targeting AN6094	AACAACCCAUAAAGTTTTAGAGCTAGAAATAGCAAGTTAAA
tRNA-gRNA-AN6094-rv2	Amplifying CRISPR fragment with PS tails targeting AN6094	AAAATCACGUACAACCAGTGCATCATCCGTGAATCGAAC
gRNA-AN6094-fwd2	Amplifying CRISPR fragment with PS tails targeting AN6094	ACGTGATTTUCCGTTTTAGAGCTAGAAATAGCAAGTTAAA
tRNA-gRNA-AN9098-rv1	Amplifying CRISPR fragment with PS tails targeting AN9098	AGCTGAGUAAATTGGATTGCATCATCCGTGAATCGAAC
gRNA-AN9098-fwd1	Amplifying CRISPR fragment with PS tails targeting AN9098	ACTCAGCUTCAGTTTTAGAGCTAGAAATAGCAAGTTAAA
tRNA-gRNA-AN9098-rv2	Amplifying CRISPR fragment with PS tails targeting AN9098	ATCCCGGTTAUATCCATATCTGCATCATCCGTGAATCGAAC
gRNA-AN9098-fwd2	Amplifying CRISPR fragment with PS tails targeting AN9098	ATAACCGGGAUGTTTTAGAGCTAGAAATAGCAAGTTAAA

tRNA-gRNA-AN3096-rv1	Amplifying CRISPR fragment with PS tails targeting AN3096	AGTAGTCGCCAUGTCCGCTGCATCATCCGTGAATCGAAC
gRNA-AN3096-fwd1	Amplifying CRISPR fragment with PS tails targeting AN3096	ATGGCGACTACUGCGTTTTAGAGCTAGAAATAGCAAGTTAAA
tRNA-gRNA-AN3096-rv2	Amplifying CRISPR fragment with PS tails targeting AN3096	AGTTCTGGCTUCCCCTTTCGTGCATCATCCGTGAATCGAAC
gRNA-AN3096-fwd2	Amplifying CRISPR fragment with PS tails targeting AN3096	AAGCCAGAACUGTTTTAGAGCTAGAAATAGCAAGTTAAA
tRNA-gRNA-AN2406-rv1	Amplifying CRISPR fragment with PS tails targeting AN2406	ATTGATGTTTTCUGCCCTTGCATCATCCGTGAATCGAAC
gRNA-AN2406-fwd1	Amplifying CRISPR fragment with PS tails targeting AN2406	AGAAAACATCAAUATGTTTTAGAGCTAGAAATAGCAAGTTAAA
tRNA-gRNA-AN2406-rv2	Amplifying CRISPR fragment with PS tails targeting AN2406	ACCTTCGCACCUTGTATATGCATCATCCGTGAATCGAAC
gRNA-AN2406-fwd2	Amplifying CRISPR fragment with PS tails targeting AN2406	AGGTGCGAAGGUAGGTTTTAGAGCTAGAAATAGCAAGTTAAA
tRNA-gRNA-AN5630-rv1	Amplifying CRISPR fragment with PS tails targeting AN5630	ATTTTCGGTGACCAUGTGCATCATCCGTGAATCGAAC
gRNA-AN5630-fwd1	Amplifying CRISPR fragment with PS tails targeting AN5630	ATGGTCACCGAAAAUGGCTGTTTTAGAGCTAGAAATAGCAAGTTAAA
tRNA-gRNAAN5630-rv2	Amplifying CRISPR fragment with PS tails targeting AN5630	AGTGAGTAAATTGUGGTGCATCATCCGTGAATCGAAC
gRNA-AN5630-fwd2	Amplifying CRISPR fragment with PS tails targeting AN5630	ACAATTTACTCACUCTTGGTTTTAGAGCTAGAAATAGCAAGTTAAA
tRNA-gRNA-AN5874-rv1	Amplifying CRISPR fragment with PS tails targeting AN5874	ATCTGGTCAGGUTGTGCATCATCCGTGAATCGAAC
gRNA-AN5874-fwd1	Amplifying CRISPR fragment with PS tails targeting AN5874	ACCTGACCAGAUATTTGTGTTTTAGAGCTAGAAATAGCAAGTTAAA
tRNA-gRNA-AN5874-rv2	Amplifying CRISPR fragment with PS tails targeting AN5874	ATCTCGGCTTGUCGGGTCTGCATCATCCGTGAATCGAAC
gRNA-AN5874-fwd2	Amplifying CRISPR fragment with PS tails targeting AN5874	ACAAGCCGAGAUATGTTTTAGAGCTAGAAATAGCAAGTTAAA
tRNA-gRNA-AN6713-rv1	Amplifying CRISPR fragment with PS tails targeting AN6713	ACGCGATTTCUACAAATGCATCATCCGTGAATCGAAC
gRNA-AN6713-fwd1	Amplifying CRISPR fragment with PS tails targeting AN6713	AGAAATCGCGUGCCAGTTTTAGAGCTAGAAATAGCAAGTTAAA
tRNA-gRNA-AN6713-rv2	Amplifying CRISPR fragment with PS tails targeting AN6713	AGTTTCCTCTUCATGCATCATCCGTGAATCGAAC
gRNA-AN6713-fwd2	Amplifying CRISPR fragment with PS tails targeting AN6713	AAGAGGAAACUGGAGGGGGTTTTAGAGCTAGAAATAGCAAGTTAAA
tRNA-gRNA-AN8849-rv1	Amplifying CRISPR fragment with PS tails targeting AN8849	AGTTGCTCTCUTTGCATCATCCGTGAATCGAAC
gRNA-AN8849-fwd1	Amplifying CRISPR fragment with PS tails targeting AN8849	AGAGAGCAACUAGCACGCAGTTTTAGAGCTAGAAATAGCAAGTTAAA
tRNA-gRNA-AN8849-rv2	Amplifying CRISPR fragment with PS tails targeting AN8849	ATTAATCACTATCUGATTGCATCATCCGTGAATCGAAC
gRNA-AN8849-fwd2	Amplifying CRISPR fragment with PS tails targeting AN8849	AGATAGTGATTAAUTTAGTTTTAGAGCTAGAAATAGCAAGTTAAA
tRNA-gRNA-AN10700-rv1	Amplifying CRISPR fragment with PS tails targeting AN10700	AGTTTCTCGGUGGATGAATGCATCATCCGTGAATCGAAC
gRNA-AN10700-fwd1	Amplifying CRISPR fragment with PS tails targeting AN10700	ACCGAGAAACUAAGTTTTAGAGCTAGAAATAGCAAGTTAAA
tRNA-gRNA-AN10700-rv2	Amplifying CRISPR fragment with PS tails targeting AN10700	ACTTCTTGAAAGCCUCTGCATCATCCGTGAATCGAAC
gRNA-AN10700-fwd2	Amplifying CRISPR fragment with PS tails targeting AN10700	AGGCTTTCAAGAAGUTTAGGTTTTAGAGCTAGAAATAGCAAGTTAAA
tRNA-gRNA-AN4663-PM-rv-2	Amplifying CRISPR fragment with PS tails targeting AN4663 E340	ATTTCGACGAUGGTCTGCATCATCCGTGAATCGAAC
gRNA-AN4663-PM-fwd-2	Amplifying CRISPR fragment with PS tails targeting AN4663 E340	ATCGTCGAAAUCGACCGTTTTAGAGCTAGAAATAGCAAGTTAAA
**Primers for Integration-vector construction**		
IS5 P1 RV	Backbone to IS5 tails	ACATGGCAGUCGGCCGCATTTAAATCCCC
IS5 P1 FW	Backbone to IS5 tails	ATCTGGACTUAGCGCGGCCGCAAATTTAAAT
IS5 pAC1 DS RV	IS5 DS with Backbone tail	AAGTCCAGAUTTACTCAATTCAGGTCTG
IS5 pAC1 US FW	IS5 US with Backbone tail	ACTGCCATGUGTCCAAGCC
IS5 pAC1 US RV	IS5 US with PacI tail	AGGGTTUAATTAAGACCTCAGCTCGCTGAGTGTGAGTCTGACT
IS5 pAC1 DS FW	IS5 DS with PacI tail	AAACCCUCAGCGCATGGCAATCAAGTCCCTGT

Ptef-FU-PacI Up	Amplifying Ptef fragment with PacI tail	GGGTTTAAUCGAGACAGCAGAATCACCG
Ptef-RU-start two	Amplifying Ptef fragment	ATGGTGAAGGUTGTGTTATGTTTTG
Ttef-FU-end A	Amplifying Ttef fragment	AGCGGACAUTCGATTTATGC
Ttef-RU-PacI Dw	Ampligying Ttef fragment with PacI tail	GGTCTTAAUGTATTGGGATGAATTTTGTATGC
P1 FW	General Backbone to IS tail	ACGCATCGGUAGCGCGGCCGCAAATTTAAAT
P1 RV	General Backbone to IS tail	AGGTCGTCCUCGGCCGCATTTAAATCCCC
AN4663-FU-Ptef	Amplifying AN4663 with Ptef tail	ACCTTCACCAUGGCGCCATTCAGGAGC
AN4663-RV-Ttef	Amplifying AN4663 with Ttef tail	ATGTCCGCUACCACCCCTCCCAGACC
AN4663-RV-mRFP v2	Amplifying AN4553 with C terminal mRFP tail	AGGCCAUCCACCCCTCCCAGACCCTAT
AN4663-FW-mRFP	Amplifying AN4553 with N terminal mRFP tail	ATGGCGCCAUTCAGGAGCAT
mRFP-RV-AN4663	Amplifying mRFP as C terminal with AN4663 tail	ATGGCGCCAUGGCGCCGGTGGAGTGG
mRFP-FW-AN4663	Amplifying mRFP as N terminal with AN4663 tail	ATGGCCUCCTCCGAGGAC
AN4663-truncv2-FU	Amplifying Truncated AN4663 without transmembrane region	ACCTTCACCAUGGCCCTCAACAACATCATCGC
ISan4663-P1-DS-RU	Amplifying ISan4663 DS with Backbone tail	ACCGATGCGUGAGGTCGAGACATTAGATATGCAAGG
ISan4663-P1-US-FW	Amplifying ISan4663 US with Backbone tail	AGGACGACCUCTTCAACGCACCATTCAGGCT
ISan4663-PacI-US-RU	Amplifying ISan4663 US with PacI tail	AGGGTTUAATTAAGACCTCAGCGTTACTATTTGGTGATCAGGCGCA
ISan4663-PacI-DS-FW	Amplifying ISan4663 DS with PacI tail	AAACCCUCAGCATGTGTTTTTCCTGACCCAGGG
**Primers for DNA insertions & gene deletion check**		
ANIS5-ChkUp-F2	Checking for insertion in IS5	CGTACTCTGTCCCACCAACC
ANIS5-ChkDw-R2	Checking for insertion in IS5	CCGCGTTATCCCTTCCTGC
AN-S53-Chk-Gap-F	Checking for insertion in IS5	CTCCTTCCTAGCCTTGCAACTCC
AN-S53-Chk-Gap-R	Checking for insertion in IS5	CCTAGCTCATTCCTCAGTCCGTC
AN-S53-Chk-Up-F	Checking for insertion in IS5	GATTGCATGGTTGGATCTGGATG
AN-S53-Chk-Dw-R	Checking for insertion in IS5	GGATGGCGAATCGTGAGCG
AN10909-Chk-FW	Checking for deletion of AN10909	GGCTTTCGCCGTGTTGTGA
AN10909-Chk-RV	Checking for deletion of AN10909	GGTGGTTGCAGTCCGTGA
AN2165-Chk-FW	Checking for deletion of AN2165	CGCAGCGGTCAAATATCCC
AN2165-Chk-RV	Checking for deletion of AN2165	GGGCGTTGGGTATTCTTTGC
AN1566-Chk-FW	Checking for deletion of AN1566	GGACCTATACCTAGTCCCCC
AN1566-Chk-RV	Checking for deletion of AN1566	AAATTGAGCGCCGCAAGC
AN0761-Chk-FW	Checking for deletion of AN0761	ACGGTCCAGGACAAACGAC
AN0761-Chk-RV	Checking for deletion of AN0761	CTTCTCTGGCTCTCACTGGG
AN0134-Chk-FW	Checking for deletion of AN0134	CGACGATTTCGCTCGAAGTG
AN0134-Chk-RV	Checking for deletion of AN0134	TAGTCCAACCCAGCTGCAT
AN4663-Chk-F	Checking for deletion of AN4663	TTGCGGGGTGAAGATGGTAG
AN4663-Chk-R	Checking for deletion of AN4663	ATGGGAATGGTGGGGTTGTT
AN3464-Chk-F	Checking for deletion of AN3464	GCGTGATGGCGTAGGATGTA
AN3464-Chk-R	Checking for deletion of AN3464	CCGTTTGGTGGAGGTCATCA
AN2405-Chk-F	Checking for deletion of AN2405	GGAGGTGGTCTCGTTTGGAG
AN2405-Chk-R	Checking for deletion of AN2405	TCAGCAGCAATTACCCGTCA
AN7375-Chk-F	Checking for deletion of AN7375	ATTTCGGTTATGCGGGGAGC
AN7375-Chk-R	Checking for deletion of AN7375	ACAATCACTTCGCCGTTCCA
AN10974-Chk-F	Checking for deletion of AN10974	GGGCTGCTCGTATCTTACCC
AN10974-Chk-R	Checking for deletion of AN10974	CCAATTGAAGTCCGTCTGCG
AN9193-Chk-F	Checking for deletion of AN9193	GCAGAGTCTCCGCTTATGGT
AN9193-Chk-R	Checking for deletion of AN9193	TCATATGCGACACGCAGGATT
AN8945-Chk-F	Checking for deletion of AN8945	AGACGGAAGTGCCGGAAAC
AN8945-Chk-R	Checking for deletion of AN8945	CTGGAAGCCGTCCAAGGTT
AN4625-Chk-F	Checking for deletion of AN4625	GAGAATAAGCCGTGCGCAGC
AN4625-Chk-R	Checking for deletion of AN4625	AGCCCTAGGCTGGAAGTAAG
AN6094-Chk-F	Checking for deletion of AN6094	AGGCGTTTCAAGGTGTTACTTCG
AN6094-Chk-R	Checking for deletion of AN6094	CCAACTCGGTAACATGATCTCCTC
AN9098-Chk-F	Checking for deletion of AN9098	CTCTTCCTCGTTCTTTTCGCGC
AN9098-Chk-R	Checking for deletion of AN9098	TTCTGCCCGTCGCGGC
AN3096-Chk-F	Checking for deletion of AN3096	CTTATCAATAGCACAGCTGAAAGAAAGGG
AN3096-Chk-R	Checking for deletion of AN3096	CAGTTGAGGAACAACGAAAGAAATGG
AN2406-Chk-F	Checking for deletion of AN2406	TCTATTGAAGAGGTGGGAGCTGG

AN2406-Chk-R	Checking for deletion of AN2406	GGTCGCGGAATGCTGGATAT
AN5630-Chk-F	Checking for deletion of AN5630	CAGTCTCCGTACTTCATGGCT
AN5630-Chk-R	Checking for deletion of AN5630	GATGAGGGGCTTGTGGAGAT
AN5874-Chk-F	Checking for deletion of AN5874	ATTTGCGCAGGGAAAGGCTAC
AN5874-Chk-R	Checking for deletion of AN5874	CAGCGCTCAACAGAGATGTTG
AN6713-Chk-F	Checking for deletion of AN6713	ACCTTTCATGTGCTCTTCGAGG
AN6713-Chk-R	Checking for deletion of AN6713	AGTCGTCCCTCTAATCACGC
AN8849-Chk-F	Checking for deletion of AN8849	ATTTTGCGACTGCTTCCCCG
AN8849-Chk-R	Checking for deletion of AN8849	ATGAGCTCCTCTCATTGCCTTC
AN10700-Chk-F	Checking for deletion of AN10700	ACGATGAGAGCTCCATCAAAGG
AN10700-Chk-R	Checking for deletion of AN10700	CTGAACGTCTTGGCCAGAAGAT
**Oligonucleotides for gene editing**		
GE-Oligo-328-329	Reparing dsb to delete the gene AN10909	ACACCACCACGATTAGTTTGCGAGTGCATAGTCAATCCGGCTAGCGGGATCCATTAGAGC
GE-Oligo-330-331	Reparing dsb to delete the gene AN2165	CCCTCTTAGAAGCTTCCAACTGCTCGGAGCCTTTCGGACGGACGAATAATGAATGATGCA
GE-Oligo-332-333	Reparing dsb to delete the gene AN1566	CGCCTAGTAACAACCGGGGCCCCGTGAACCGAGACTGTATTAGTAGATGTTCTGCGCATA
GE-Oligo-338-339	Reparing dsb to delete the gene AN0761	CACATCTCGGTGATACGGATCGCTCAGGGAAACATGGACGACCGAGGGTGAGCGAAAAGC
GE-Oligo-340-341	Reparing dsb to delete the gene AN0134	GTCTTCTTCCGTTTTATATCAGGTGTCAGTCGGAGAAAGTCTGCTTTAACTCGTTCCGGA
GE-Oligo-AN4663	Reparing dsb to delete the gene AN4663	TCTGTCTGCGCCTGATCACCAAATAGTAACATGTGTTTTTCCTGACCCAGGGCGAAAGTG
GE-Oligo-AN3464	Reparing dsb to delete the gene AN3464	CTAAAACAAACGACCAGAAGATATACAACGAGACCTGAGTTCTGCGATATGCTATGGAAT
GE-Oligo-AN2405	Reparing dsb to delete the gene AN2405	TGTTTTGTATAGAAGACGGATACGCTCACATGCCGCTATATCCGCTGCTCGTCATAGTAT
GE-Oligo-AN7375	Reparing dsb to delete the gene AN7375	GGGATCCTAGAGGTCAAAAATCAATCGACGGCTGCTGTTGGTGAATCTGTTATCAGAAGT
GE-Oligo-AN10974	Reparing dsb to delete the gene AN10974	AAGCTTCCGTTCGTCTGCCAAATAATCAACTCATGTAACAAAACCATATTAACTCATGGG
GE-Oligo--AN9193	Reparing dsb to delete the gene AN9193	AACAAGCCTTCAAACCTCCTAATAAACTCCCTCGCCGTACCAACCACTAGTTATATTCGG
GE-Oligo--AN8945	Reparing dsb to delete the gene AN8945	TCTTTTGATTTTACTATTTTGTCGGTCGCAAGCTGGCTTATTGTGGCTTCAGTCATCGTA
GE-Oligo--AN4625	Reparing dsb to delete the gene AN4625	AGCCTTCATTCTAATCCCAATTTATCGAGAACAGAACTCTACCGTACGTGCGCCAGATTT
GE-Oligo--AN6094	Reparing dsb to delete the gene AN6094	CCATCATTTATCTACAAGCTTGTCTACGAATCTCGCGTCTGGTTGTACGTGATTTTCCGG
GE-Oligo--AN9098	Reparing dsb to delete the gene AN9098	AGCTACAGAGAATCCAATTTACTCAGCTTCACGAAGTGATATATGATTCGGGAAAAAGCG
GE-Oligo--AN3096	Reparing dsb to delete the gene AN3096	GACTGGGTTTTTTCCCCCGCAGTAGTCGCCATATTAAGCTTTAAAATAAAAGTTTTGCTT
GE-Oligo-AN2406	AN2406	ATCCCCTCGCACAGGGCAGAAAACATCAATAGGTGATTCGGAAGAGTTGTATATACGGAC
GE-Oligo-AN5630	AN5630	ATTTGTCAGTACCAGGCATGGTCACCGAAAGTAAATTGTGGCGGACTTGCAATCTGCTCT
GE-Oligo-AN5874	Reparing dsb to delete the gene AN5874	AGAAAATACTTCAAGCCTTGACAGGCGATAGCGCGATGAATGGTGAAAATATCACCAAAC
GE-Oligo-AN6713	Reparing dsb to delete the gene AN6713	GCAATCACTTGCTGATATAGCTATTGCACCACTACTAGGTAGCTTTTTTGTCCGTATCAC
GE-Oligo-AN8849	Reparing dsb to delete the gene AN8849	TCCACATACGGAAGAGAGCAACTAGCACGCTTTATGGAAATACATGTATGCATAACAGAT
GE-Oligo-AN10700	Reparing dsb to delete the gene AN10700	CTTCCCAAACGTTCATCCACCGAGAAACTAGTTGACGATATGTTTTGATTTGATACATAA
GE-oligo AN4663 PM E340A	Reparing dsb and mutate AN4663 E340A	GAATCGACACGACCATCGTCGCCATCGACCCGGTTGTCCACAAGT
**Primers for sequencing**		
pFC902sgRNA-Seq-F	To sequence the tRNA, protospacer, gRNA and tRNA	CATCGCATCCTCATGAAGTC
AN4663-PM-Seq-Fw	To sequence for checking the point mutation AN4663 E340A	GTCATGAGATGCGACCACAG
AN4663-PM-Seq-Rv	To sequence for checking the point mutation AN4663 E340A	TCGCCGGCATAATTCTTCAGTC
Ptef1-SeqInsert-F	To sequence for insertion using Ptef as promoter	CCTCGCTTCTCTCCTCCATC

Ttef1-SeqInsert-R	To sequence for insertion using Ttef as terminator	CAAGGTACACGCAAAACGAGGTAC
**PS name/number**	**Target**	**Sequence (5’ à 3’)**
**Target sequences involved in gene editing (Protospacers)**		
PS328	AN10909	CGAGTGCATAATGTCCTTCA
PS329	AN10909	CCCACCACCTGCATTAGTTC
PS330	AN2165	AAATTTCCATGAGCAGATTC
PS331	AN2165	CGCGAAGCAATGAACTGCTC
PS332	AN1566	TAAGGACAACTGGTCATCCG
PS333	AN1566	GGCTAGGGTATGTTAGGCTT
PS338	AN0761	ATCGGGAGGTGCTGAGATGA
PS339	AN0761	TCACCCTTCTGCCCTATTTG
PS340	AN0134	CAGTATGGAATCTGAAAGCG
PS341	AN0134	TACACTCAAGCATTAAGGAT
PS-AN4663-1	AN4663	GCTACCAAGAAGCTGGTAGT
PS-AN4663-2	AN4663	GATTATGAGGACTGTTTTGC
PS-AN3464-1	AN3464	ACGATGGCGTGGTACTGTTC
PS-AN3464-2	AN3464	TCAGTACCGCATTCGGCGGT
PS-AN2405-1	AN2405	CGCTCACAATGGCATCTGCA
PS-AN2405-2	AN2405	AGCGGATATAGCGGCACTAC
PS-AN7375-1	AN7375	AAGAAATCAGTCGAGCTCTC
PS-AN7375-2	AN7375	AACGGTGTTGCCTCTCAGTT
PS-AN10974-1	AN10974	TTGACATAGTCGACATTCGC
PS-AN10974-2	AN10974	TGAGTTTGGAAGCGGTTCCG
PS-AN9193-1	AN9193	TGCAGCGTCAGCCATGGTCA
PS-AN9193-2	AN9193	GCAGGTTCAATGTTGTGGTC
PS-AN8945-1	AN8945	TATTTTGTCGGTCGCAATGG
PS-AN8945-2	AN8945	GGCAGAAGCCGGAGTAGAGC
PS-AN4625-1	AN4625	GATTCAAGACACCTACTCCG
PS-AN4625-2	AN4625	CAGATCTATGCTGTCAAGGG
PS-AN6094-1	AN6094	CCTTCACAACAACCCATAAA
PS-AN6094-2	AN6094	CAGGTTGTACGTGATTTTCC
PS-AN9098-1	AN9098	ATCCAATTTACTCAGCTTCA
PS-AN9098-2	AN9098	GATATGGATATAACCGGGAT
PS-AN3096-1	AN3096	GCGGACATGGCGACTACTGC
PS-AN3096-2	AN3096	CGAAAGGGGAAGCCAGAACT
psAN2406-1	AN2406	AGGGCAGAAAACATCAATAT
psAN2406-2	AN2406	TATACAAGGTGCGAAGGTAG
PS-AN5630-1	AN5630	CATGGTCACCGAAAATGGCT
PS-AN5630-2	AN5630	CCACAATTTACTCACTCTTG
PS-AN5874-1	AN5874	CAACCTGACCAGATATTTGT
PS-AN5874-2	AN5874	GACCCGACAAGCCGAGATAT
PS-AN6713-1	AN6713	TTTGTAGAAATCGCGTGCCA
PS-AN6713-2	AN6713	TGAAGAGGAAACTGGAGGGG
PS-AN8849-1	AN8849	AAGAGAGCAACTAGCACGCA
PS-AN8849-2	AN8849	ATCAGATAGTGATTAATTTA
PS-AN10700-1	AN10700	TTCATCCACCGAGAAACTAA
PS-AN10700-2	AN10700	GAGGCTTTCAAGAAGTTTAG
PS-AN4663-point-mutation-2	AN4663-E340	GACCATCGTCGAAATCGACC
**Primers for *K. phaffii***		
**Fw-PmeI**		AAACGCTGTCTTGGAACCTA
**Rv-PmeI**		AAACTGTCAGTTTTGGGCCAT
**Fw_SapI_AN4663**		CGGGTTTGCTCTTCGCATACAACGACCGCACTTAATAATATCATCGCTG
**Rv_SapI_AN4663**		AAACCCGGGGCTCTTCGTTACCAGCCCTCCCAGACGCG
**FW-NativeSP_LsAA9A**		ACGGCACTUTCCTTCGTCGCGTCAGCCGCGGCTCATACCCTCGTCTGGGGC
**RV-NativeSP_LsAA9A**		AAGTGCCGUGAGCCCGAGGATGGAGTACTTCATCGTTTGGATCCTTCGAATAATTAGTTGTTTTTTGATC
**FW-USER-AN4663-episomal**		ATTGAACAACUATTTCGAAACGATGGCGCCTTTTCGTTCAATTTATG
**RV-USER-AN4663-GAP-episomal**		AGTTGTTCAAUTGATTGAAATAGGGACAAATAAATTAAATTTAAAGTC

**Fw-AOX1**	GACTGGTTCCAATTGACAAGC
**Rv-AOX1**	GCAAATGGCATTCTGACATCC

Supplementary Note 1: Native DNA sequence of LsAA9A used for heterologous expression in *K. phaffii*.

Native^{SP}-LsAA9A

```
          1 atgaagtact ccatcctcgg gctcacggca ctttccttcg tcgcgtcagc
cgcggctcat
         61 accctcgtct ggggcgtatg ggtcaacggc gttgatcaag gagatgggag
gaacatctac
        121 atcaggtctc ccccgaacaa taaccccgtc aagaacctga cttcgccgga
tatgacgtgc
        181 aatgtcgaca atcgcgttgt gcccaagagc gtgccagtca acgctggtga
tacgcttact
        241 ttcgaatggt accacaacac acgagacgac gatatcatcg catcttctca
tcacggaccc
        301 atcgccgtat acatcgcacc tgccgcttcg aacggtcaag ggaacgtttg
ggtgaagctg
        361 ttcgaggacg cctacaacgt caccaatagc acttgggctg tcgatcgtct
catcactgct
        421 catggacaac actccgttgt ggtcccccat gttgcacctg gagactacct
atttagagct
        481 gagatcattg cgctacatga ggcagattct ctgtactccc agaaccctat
ccgtggcgcg
        541 caattttata tctcgtgcgc tcaaatcacc atcaactctt ccgatgactc
gacacctctc
        601 cccgctggtg ttcccttccc cggtgcatac accgactcca cacccggtat
tcaattcaat
        661 atctacacta cccccgcgac aagctacgtt gctccccctc ccagcgtatg
gtctggagcg
        721 ttgggtgggt cgattgctca ggtcggagat gcttctctcg ag
```
//

Supplementary Note 2: Native DNA sequence of TfAA10A used for heterologous expression in *K. phaffii*.

TfAA10A

```
        1 catgggtcgg tcatcaaccc cgcgacccgt aactacggtt gctggctgcg
ttggggccac
       61 gaccacctca accccaacat gcagtacgaa gaccccatgt gctggcaggc
ctggcaggac
      121 aaccccaacg ccatgtggaa ctggaacggc ctgtaccgcg actgggtcgg
cggcaaccac
      181 cgggctgccc tccccgacgg ccagctgtgc agcggtggcc tcaccgaagg
cggccgctac
      241 cgctccatgg acgccgtagg cccgtggaag accaccgacg tcaacaacac
cttcaccatc
      301 cacctgtacg accaggccag ccacggcgca gactacttcc tggtctacgt
caccaagcag
      361 ggcttcgacc cgaccaccca gccgctgacc tgggacagcc tggaactggt
gcaccagacc
      421 ggcagctacc ccccggccca gaacatccag ttcacggtcc acgcccccaa
ccgcagcggc
      481 cgccacgtgg tcttcaccat ctggaaggcc tcgcacatgg accagaccta
ctacctgtgc
      541 agcgacgtga acttcgtc
```

Supplementary Note 3: DNA sequence AN4663 in pBGP1

Codon variant AN4663

```
   1 atggcgcctt ttcgttcaat ttatgagaaa gatgccacca aaaagcttgt tgtaggagca

  61 gcgttgcttg tgctggctgc gttttatagt tatgtatttc tgctgactct ggcgcctgta

 121 tatggttcta ctccttccca tatctttcac ggctatggag tcggtatcgc gggtgtagct

 181 ggctggtttt cgaaggacat tgtggatcgc gtaagcggtc gtaaagcaat ctatgcaatt

 241 ccagttttgg cgttcttttt accagtcgtt caatactttg tcagtcagca gtcatctgca

 301 cttggcaatc ctgcggggcc aatcttcaca gaggtattgg ctctgtaccc tctggtcttg

 361 ttatcagttg cttgtgcggg gaagctggtc caggccggtc ttaacttgca acgccatgga

 421 gacttggtag ctgaacacat tcctttactt gggtcgtacg ttatttattc tgcaggggag

 481 catcttatca aggccttctt atctcgcttc atcggctcca ccgtactttt gtcacgcgct

 541 ggcttacaga ttcttatcgc aatttctac gctgccgccg ttccttccaa ggcgttactt

 601 ctggccattc cagcgttctt attctcagtc acatcgaaca cccaccttcc tttgggccac

 661 acaacgaccg cacttaataa tatcatcgct gatgacggct tcgcacttgt tgcacgccaa

 721 gacagcacta ccgggtacat ttcagtcttg gataaccttg aggatgggtt tcgcgtaatg

 781 cgctgtgacc acagtttact gggaggtcaa tggatcaaaa aacgtcccaa ttatactcct

 841 ccagccgtaa aggaccccat ctatgcagta ttcacaatgt tggaggcagt gcgcttggtc

 901 gaaacggctc acggtatccc ccgtgctgat gcgggctcca acgcgcttgt aatcggcttg

 961 gggatcggca ctacccctgg cgctttgatt agtcacggaa ttgacaccac gattgtcgaa

1021 attgaccctg ttgtccacaa atatgccctt caatattttg accttcctga aaaccacaca

1081 cctatcattg aggacgcacg cgcttttgta cagcgttcgc gtaatgctcc acaacccaag
```

1141 cagtacgatt acattgtgca tgacgtgttc actggcggag ctgagccggt agagttgttc

1201 acctatgagt tcatctcggg cctgcatgcg cttttgaaag acgatggagt tatcgccatt

1261 aactacgcag gggatatttc cttatattca acagctctga gcatccgcac aatcaaaagc

1321 atttttccca cctgtcgctt gttccgcgag gctgccgccc cggagatcgg accggatttt

1381 acgaatatgg tcattttctg cacgaaatcg cgtggtgcac cgattacatt ccgcgatccc

1441 gtaccggaag atttcttggg aagccgcttt cgttctcgtt accttgttcc aaaacatgag

1501 gtagatgccg cgcaattcga caacgtcggg ttggaagacg gtcctcaggg acatggccgc

1561 cgcgtgctgg ttgacaaaga ggtcggtcgt ttacacaaat atcaggaccg ttccgcactg

1621 gagcattggg gaattatgcg taccgtcttg ccagatcgcg tctgggaggg ctgg

//

Supplementary References

1. C. Hernández-Rollán, K. B. Falkenberg, M. Rennig, A. B. Bertelsen, J. Ø. Ipsen, S. Brander, D. O. Daley, K. S. Johansen, M. H. H. Nørholm, LyGo: A Platform for Rapid Screening of Lytic Polysaccharide Monooxygenase Production. ACS Synthetic Biology. 10, 897–906 (2021).

2. C. C. Lee, T. G. Williams, D. W. S. Wong, G. H. Robertson, An episomal expression vector for screening mutant gene libraries in *Pichia pastoris*. Plasmid. 54, 80–85 (2005).

3. C. S. Nødvig, J. B. Nielsen, M. E. Kogle, U. H. Mortensen, A CRISPR-Cas9 System for Genetic Engineering of Filamentous Fungi. PLOS ONE. 10, e0133085 (2015).
