# Supplementary material for: A seven-transmembrane methyltransferase catalysing N-terminal histidine methylation of lytic polysaccharide monooxygenases
Source: Nat Commun. 2023 Jul 14;14:4202. doi: 10.1038/s41467-023-39875-7 (PMC10349129; doi:10.1038/s41467-023-39875-7)
Supplement: Supplementary file 3 — Description of Additional Supplementary Files [file 41467_2023_39875_MOESM3_ESM.pdf]

Description of Additional Supplementary Files for “A seven-transmembrane methyltransferase catalysing N-terminal histidine methylation of lytic polysaccharide monooxygenases”

File Name: Supplementary Data 1
Description: This supplementary data contains protein group identifications from the large-scale proteomics analysis of *Aspergillus nidulans*. Three major proteomics analysis were performed (TMT, LFQ, Deep proteome profiling) and included in the dataset.

File Name: Supplementary Data 2
Description: This supplementary data contains histidine methylation sites identified using MaxQuant from the different proteomics analysis of *Aspergillus nidulans*. Contaminant and reverse hits were removed from the list.

File Name: Supplementary Data 3
Description: This supplementary data contains protein annotations of the *Aspergillus nidulans* (also known as *Emericella nidulans*) FASTA from different sources including gene ontology, pFam, KEGG, and InterPro. Methyltransferases are annotated as "+" in the "MTase" column.

File Name: Supplementary Data 4
Description: This supplementary data contains methyltransferases candidates which were short listed based on proteomics differential abundance analysis.

File Name: Supplementary Data 5
Description: This supplementary data contains quantification of the Q5B428 N-terminal histidine methylated HTVIVYPGYR for the different methyltransferase knockouts. The data was analyzed using Skyline output was used to produce Figure 2, 4 and Figure S1, S3.

File Name: Supplementary Data 6
Description: This supplementary data contains quantification of LsAA9A n-terminal peptide with different signal peptides and the N-terminal methylation stoichiometry of LsAA9A and TfLPMO.
